# Natural Xanthones and Skin Inflammatory Diseases: Multitargeting Mechanisms of Action and Potential Application

**DOI:** 10.3389/fphar.2020.594202

**Published:** 2020-12-03

**Authors:** Natalie Vivien Gunter, Soek Sin Teh, Yang Mooi Lim, Siau Hui Mah

**Affiliations:** ^1^ School of Biosciences, Taylor’s University, Subang Jaya, Malaysia; ^2^ Engineering and Processing Division, Energy and Environment Unit, Malaysian Palm Oil Board, Kajang, Malaysia; ^3^ Centre for Cancer Research, Faculty of Medicine and Health Sciences, Universiti Tunku Abdul Rahman, Kajang, Malaysia; ^4^ Department of Pre-Clinical Sciences, Faculty of Medicine and Health Sciences, Universiti Tunku Abdul Rahman, Kajang, Malaysia; ^5^ Centre for Drug Discovery and Molecular Pharmacology, Faculty of Health and Medical Sciences, Taylor’s University, Subang Jaya, Malaysia

**Keywords:** acne, anti-inflammatory, antioxidant, atopic dermatitis, skin cancer, psoriasis

## Abstract

The pathogenesis of skin inflammatory diseases such as atopic dermatitis, acne, psoriasis, and skin cancers generally involve the generation of oxidative stress and chronic inflammation. Exposure of the skin to external aggressors such as ultraviolet (UV) radiation and xenobiotics induces the generation of reactive oxygen species (ROS) which subsequently activates immune responses and causes immunological aberrations. Hence, antioxidant and anti-inflammatory agents were considered to be potential compounds to treat skin inflammatory diseases. A prime example of such compounds is xanthone (xanthene-9-one), a class of natural compounds that possess a wide range of biological activities including antioxidant, anti-inflammatory, antimicrobial, cytotoxic, and chemotherapeutic effects. Many studies reported various mechanisms of action by xanthones for the treatment of skin inflammatory diseases. These mechanisms of action commonly involve the modulation of various pro-inflammatory cytokines such as interleukin (IL)-1β, IL-6, IL-8, and tumor necrosis factor α (TNF-α), as well as anti-inflammatory cytokines such as IL-10. Other mechanisms of action include the regulation of NF-κB and MAPK signaling pathways, besides immune cell recruitment via modulation of chemokines, activation, and infiltration. Moreover, disease-specific activity contributed by xanthones, such as antibacterial action against *Propionibacterium acnes* and *Staphylococcus epidermidis* for acne treatment, and numerous cytotoxic mechanisms involving pro-apoptotic and anti-metastatic effects for skin cancer treatment have been extensively elucidated. Furthermore, xanthones have been reported to modulate pathways responsible for mediating oxidative stress and inflammation such as PPAR, nuclear factor erythroid 2-related factor and prostaglandin cascades. These pathways were also implicated in skin inflammatory diseases. Xanthones including the prenylated α-mangostin (**2**) and γ-mangostin (**3**), glucosylated mangiferin (**4**) and the caged xanthone gambogic acid (**8**) are potential lead compounds to be further developed into pharmaceutical agents for the treatment of skin inflammatory diseases. Future studies on the structure-activity relationships, molecular mechanisms, and applications of xanthones for the treatment of skin inflammatory diseases are thus highly recommended.

## Introduction

Skin inflammatory diseases are mediated by multiple mechanisms that induce chronic inflammation (Pleguezuelos-Villa et al., 2019). For instance, the pathogenesis of skin inflammatory diseases such as atopic dermatitis (AD), acne, psoriasis and skin malignancies have been extensively associated with immunological alterations in cytokine expression and immune cell activity. Inflammatory responses were additionally linked to the generation of oxidative stress by external aggressors such as ultraviolet (UV) radiation and xenobiotics that also disrupts the normal intracellular redox state and cellular protein structures (Li et al., 2012; Cidade et al., 2017). These inflammatory responses in skin inflammatory diseases are commonly modulated through NF-κB, MAPK and PI3K/Akt signaling pathways which contribute to the activation of immune cells and production of pro-inflammatory cytokines such as interleukin (IL)-1β, IL-6, IL-8 and tumor necrosis factor α (TNF-α) (Wang et al., 2017; Guo et al., 2019). Thus, the potential lead compounds that possess both anti-inflammatory and antioxidant activities such as xanthones have received great attention for the treatment for skin inflammatory diseases (Pleguezuelos-Villa et al., 2019).

Xanthones are versatile scaffolds consisting of a tricyclic xanthene-9-one structure (**1**) and are commonly found in higher plants, fungi, and lichens (Aye et al., 2020). Various plants have been reported to be rich in naturally occurring xanthones, including *Garcinia mangostana* L. (Im et al., 2017), *G. cowa* Roxb. ex Choisy (Auranwiwat et al., 2014), *G. hanburyi* Hook.f. (Xu et al., 2015), *Mangifera indica* L. (Garrido et al., 2004), *Hypericum oblongifolium* Wall. (Ali et al., 2011), *Tripterospermum lanceolatum* (Hayata) H. Hara ex Satake (Hsu et al., 1997), *Mesua beccariana* (Baill.) Kosterm., *M. ferrea* L. (Teh et al., 2017), *Iris sibirica* L. (Tikhomirova and Ilyicheva, 2020), *I. adriatica* Trinajstic ex Mitic (Alperth et al., 2018), *Calophyllum inophyllum* L. (Mah et al., 2019) and *C. soulattri* Burm.f. (Mah et al., 2019). Furthermore, plants of the genera *Calophyllum*, *Cratoxylum*, *Garcinia*, *Gentiana*, *Hypericum* and *Swertia* were proposed to possess great developmental prospect due to their rich content of xanthones (Ruan et al., 2017). This class of compounds possess a wide range of biological activities including anti-inflammatory (Teh et al., 2017; Mah et al., 2019; Aye et al., 2020; Ng et al., 2020), antioxidant (Wang et al., 2018a), antimicrobial (Koh et al., 2013; Auranwiwat et al., 2014), chemotherapeutic (Beninati et al., 2014) and chemopreventive (Purnaningsih et al., 2018) activities. Thus, xanthones are promising lead compounds for treating skin inflammatory diseases.

**FIGURE 1:**
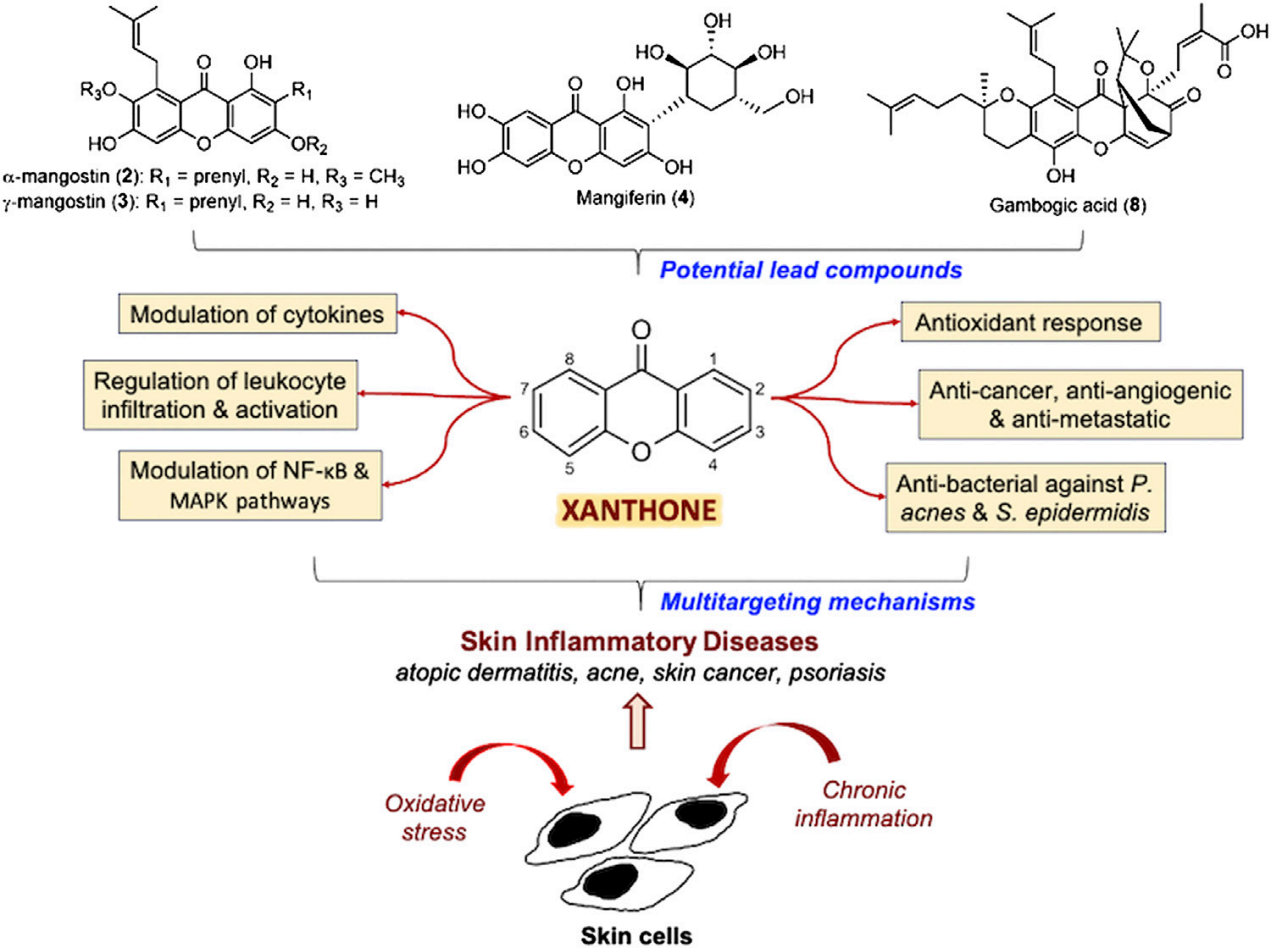
Summary of the multitargeting mechanisms of action of xanthones for skin inflammatory diseases.

**FIGURE 2:**
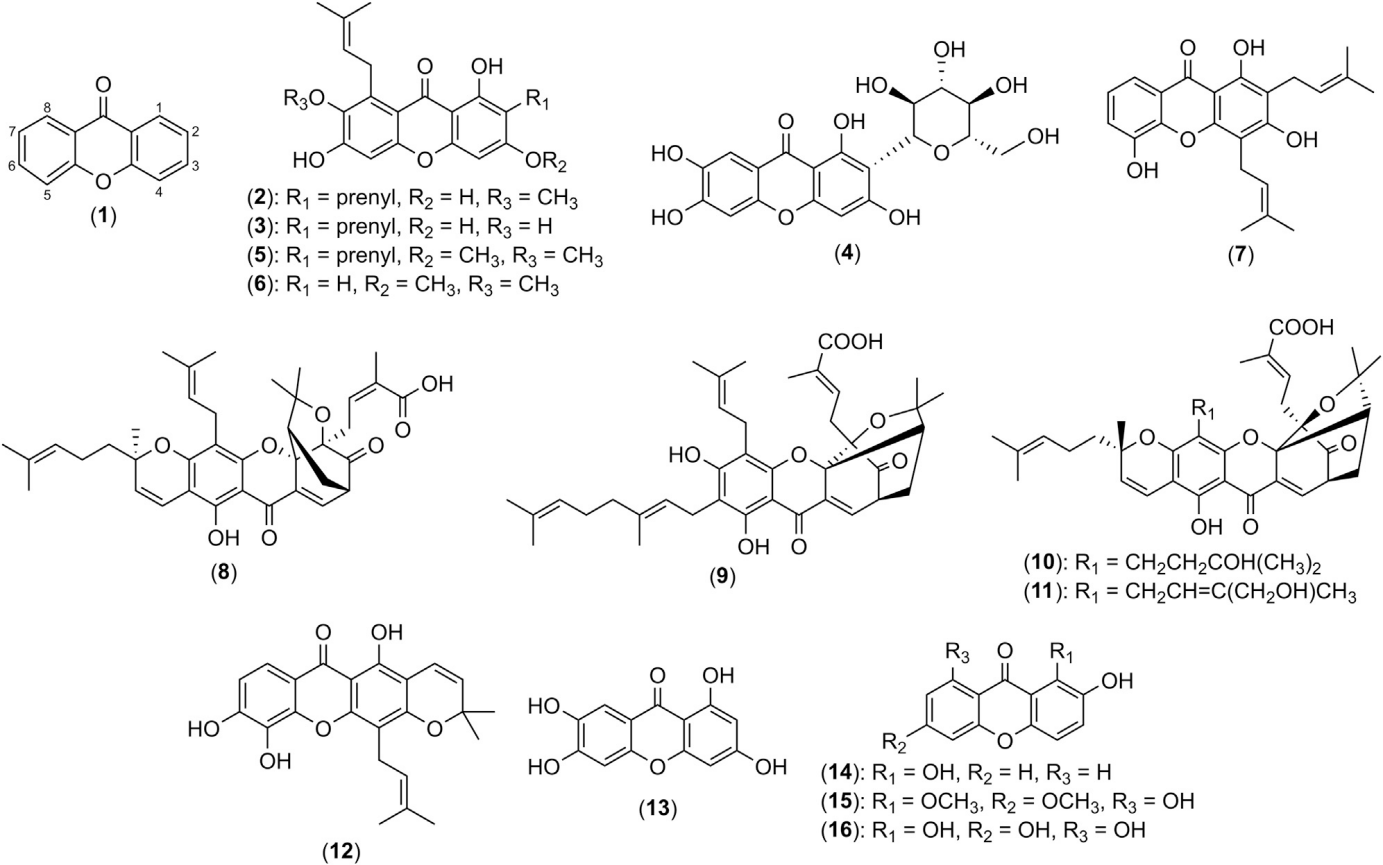
Structures of xanthene-9-one (**1**), α-mangostin (**2**), γ-mangostin (**3**), mangiferin (**4**), β-mangostin (**5**), garcicowanone A (**6**), 8-deoxygartanin (**7**), gambogic acid (**8**), gambogenic acid (**9**), 33-hydroxyepigambogic acid (**10**), 35-hydroxyepigambogic acid (**11**), xanthone V_1_ (**12**), norathyriol (**13**), 1,2-dihydroxyxanthone (**14**), gentiacaulein (**15**) and norswertianin (**16**).

Many reviews to date discuss on various biological activities of xanthones (Ibrahim et al., 2016; Aizat et al., 2019; Feng et al., 2020; Ong et al., 2020) with a recent review discussing the modulatory ability of xanthones on macrophage function and associated inflammatory diseases (Ng and Chua, 2019). The reviews on the potential of xanthones as anti-inflammatory agents for skin diseases are limited and no review has been focused extensively on their mechanisms of action in the context of skin inflammatory diseases. Thus, in this review, we focused on the studies of naturally occurring xanthones within the past two decades for the treatment of skin inflammatory diseases, particularly atopic dermatitis, acne, psoriasis, and skin cancers, while simultaneously highlighting the mechanisms of action and signaling pathways involved. A summary of the mechanisms of action for xanthone derivatives with anti-inflammatory, anti-bacterial, chemotherapeutic and cytoprotective effects are summarized in Table 1; Figure 1. The basic structure of xanthone is presented in Figure 2, together with its naturally available derivatives that exhibit these biological activities. The potential applications of xanthones with regards to cellular pathways implicated in skin inflammatory diseases are also included in this review.

**TABLE 1:** Xanthones and their mechanism of action for treatment of skin inflammatory diseases.

Disease	Xanthone	Mechanism of Action	References
Atopic dermatitis	Xanthene-9-one (**1**)	Reduced serum levels of histaminein DNFB-induced mice	Aye et al. (2020)
		Suppressed histamine and TSLP release by PMA-CI-stimulated HMC-1 mast cells	
		Reduced mRNA expression of IL-1β, IL-6, IL-8, TARC and MDC in mice	
		Reduced serum levels of IgE	
		Reduced population of mast cells in skin lesions	
		Inhibited phosphorylation of IκBα and NF-κB in HaCaT cells	
	α-mangostin (**2**)	Reduced skin lesions and hyperplasia in DNFB-induced mice	Aye et al. (2020)
		Anti-itching effects by decreasing production of NGF and nerve extensions	Nakatani et al. (2002)
		Antagonist of H1 receptors by competitive inhibition	Chairungsrilerd et al. (1996a), Chairungsrilerd et al. (1996b)
		Inhibited mRNA expression of TSLP and IFN-γ	Higuchi et al. (2013)
		Suppressed IgE production by stimulated splenic B cells	Jang et al. (2012), Higuchi et al. (2013)
		Inhibited allergen-IgE-induced mast cell activation	Jang et al. (2012)
		Reduced eosinophil population in skin of NC/Tnd mice by suppressing PI3K recruiting and activating activity	Higuchi et al. (2013)
		Inhibited NF-κB and MAPK pathways	Jang et al. (2012), Xu et al. (2018)
	γ-mangostin (**3**)	Antagonist of H1 receptor	Sukma et al. (2011)
		Suppressed IgE production by stimulated splenic B cells	Jang et al. (2012), Higuchi et al. (2013)
		Inhibited allergen-IgE-induced mast cell activation	Jang et al. (2012)
		Reduced eosinophil population in skin of NC/Tnd mice by suppressing PI3K recruiting and activating activity	Higuchi et al. (2013)
	Mangiferin (**4**)	Reduced serum levels of IL-4, IL-5, IL-13, IL-17A and TNF-α	Guo et al. (2014), Zhao et al. (2017), Yun et al. (2019)
		Increased serum levels of IL-10 and TGF-β	Guo et al. (2014), Zhao et al. (2017), Yun et al. (2019)
		Reduced secretion of IL-1β, IL-6 and iNOS by RAW264.7 macrophages	Zhao et al. (2017)
		Decreased Th9 and Th17, and increased Treg subsets in the spleen	Yun et al. (2019)
		Restored imbalanced Th1/Th2 cell ratio	Guo et al. (2014)
		Reduced inflammatory cell infiltration and eosinophil population in the skin	Guo et al. (2014), Zhao et al. (2017), Yun et al. (2019)
		Inhibited phosphorylation of IκB and reduced levels of NF-κB2	Zhao et al. (2017)
Acne	α-mangostin (**2**)	Bacteriostatic and bacteriocidal activity on Gram-positive bacteria	Koh et al. (2013), Tatiya-Aphiradee et al. (2016), Sivaranjani et al. (2019)
		Antibacterial activity against *P. acnes* and *S. epidermidis*	Auranwiwat et al. (2014), Sivaranjani et al. (2017), Xu et al. (2018)
		Targeted and disrupted cellular membrane integrity, causing rapid loss of intracellular components	Koh et al. (2013)
		Inhibited mRNA expression of IL-1β, IL-6 and TNF-α in *P. acnes*-induced HaCaT cells	Xu et al. (2018)
		Inhibited activation of NF-κB and MAPK pathways by suppressing phosphorylation of IκB, p65, p38, ERK and JNK	Xu et al. (2018)
		Inhibited lipase activity of *P. acnes*	Xu et al. (2018)
	γ-mangostin (**3**)	Antibacterial activity against *P. acnes*	Xu et al. (2018)
		Inhibited mRNA expression of IL-1β, IL-6 and TNF-α in P. acnes-induced HaCaT cells	
		Inhibited activation of NF-κB and MAPK pathways by suppressing phosphorylation of IκB, p65, p38, ERK and JNK	
		Inhibited lipase activity of *P. acnes*	
	β-mangostin (**5**)	Antibacterial activity against *S. epidermidis*	Auranwiwat et al. (2014)
	Garcicowanone A (**6**)	Antibacterial activity against *S. epidermidis*	Auranwiwat et al. (2014)
Skin cancer and melanoma	α-mangostin (**2**)	Inhibited *in vitro* cell proliferation, adhesion and invasion of SK-MEL-28, B16-F10 and A375 cells	Wang et al. (2011), Beninati et al. (2014)
		Inhibited tumor incidence and hyperplasia in DMBA-TPA-induced mice	Wang et al. (2017)
		Increased caspase-3 activity and caused loss of mitochondrial membrane potential	Wang et al. (2011)
		Increased expression of Bax, Bad and caspase-3	Wang et al. (2017)
		Downregulation of Bcl-2 and Bal-xL	Wang et al. (2017)
		Induced cell differentiation and aggregation in B16-F10 cells	Beninati et al. (2014)
		Anti-metastatic effect by reducing MMP-1 and MMP-9 activity	Beninati et al. (2014), Im et al. (2017)
		Decreased phosphorylation of PI3K, Akt and TOR proteins	Wang et al. (2017), Xia and Sun (2018)
		Induced ROS generation	Xia and Sun (2018)
		Downregulated IL-1β, IL-4 and IL-18 in DMBA-TPA-induced skin tumorigenesis in mice	Im et al. (2017), Wang et al. (2017)
		Upregulated IL-10 in DMBA-TPA-induced skin tumorigenesis in mice	Wang et al. (2017)
		Chemoprevention by photoprotective and antioxidative activity	Im et al. (2017), Purnaningsih et al. (2018)
	γ-mangostin (**3**)	Inhibited *in vitro* cell proliferation of SK-MEL-28 cells	Wang et al. (2011)
		Induced apoptosis by causing the loss of mitochondrial membrane potential	
		Chemoprevention by photoprotective and antioxidative activity	Im et al. (2017), Purnaningsih et al. (2018)
	Mangiferin (**4**)	Anti-metastatic and anti-angiogenic effect by selectively inhibiting the expression of *VEGFR2*, *MMP-19* and *PGF* genes	Delgado-Hernández et al. (2019)
		Selectively inhibited NF-κB genes including *IL-6*, *TNF*, *IFN-γ* and *CCL2*	Delgado-Hernández et al. (2019)
		Increased activity of SOD in skin of UVB-irradiated SKH-1 mice	Petrova et al. (2011)
		Chemoprevention by photoprotective and antioxidative activity	Magcwebeba et al. (2016a), Magcwebeba et al. (2016b)
	β-mangostin (**5**)	Chemoprevention by photoprotective and antioxidative activity	Im et al. (2017), Purnaningsih et al. (2018)
	8-deoxygartanin (**7**)	Inhibited *in vitro* cell proliferation of SK-MEL-28 cells	Wang et al. (2011)
		Induced apoptosis by causing the loss of mitochondrial membrane potential	
	Gambogic acid (**8**)	Inhibited cell proliferation and viability in B16-F10 and A375 cells	Zhao et al. (2008), Xu et al. (2009)
		Decreased *in vivo* expression of Akt1 and Akt2	Liang et al. (2018)
		Induced apoptosis by increasing Bax/Bcl-2 ratio and activation of caspase-3	Xu et al. (2009)
		Modulated mitochondrial p66shc/ROS-p53/Bax signaling	Liang and Zhang (2016)
		Modulated miR-199a-3p/ZEB1 signaling	Liang et al. (2018)
		Anti-metastatic effect by downregulating α4 integrin and fibronectin adhesion	Zhao et al. (2008)
	Gambogenic acid (**9**)	Induced apoptosis via PI3K/Akt/mTOR signaling pathway	Cheng et al. (2014)
	33-hydroxyepigambogic acid (**10**)	Cytotoxic activity against SK-MEL-28 cells	Xu et al. (2015)
		Induced cell cycle arrest at S or G2/M phase	
		Induced apoptosis by increasing the activity of caspase-3/7	
	35-hydroxyepigambogic acid (**11**)	Cytotoxic activity against SK-MEL-28 cells	Xu et al. (2015)
		Induced cell cycle arrest at S or G2/M phase	
		Induced apoptosis by increasing the activity of caspase-3/7	
	Xanthone V_1_ (**12**)	Induced cell cycle arrest at S phase	Kuete et al. (2011)
		Induced apoptosis by increasing activation of caspase-3/7	
	Norathyriol (**13**)	Inhibited skin carcinogenesis in SKH-1 hairless mice exposed to solar UV	Li et al. (2012)
		Induced cell cycle arrest at G2/M phase by increasing cyclin B1 and phosphorylated Cdk 1	
		Inhibited activation of MAPK and NF-κB cascades by competitively inhibiting ERK2 kinases	
	Gentiacaulein (**15**)	Chemoprevention by photoprotection and quenching of excited riboflavin	Hirakawa et al. (2005)
	Norswertianin (**16**)	Chemoprevention by photoprotection and quenching of excited riboflavin	Hirakawa et al. (2005)
Psoriasis	Mangiferin (**4**)	Cytoprotective effects on H_2_O_2_^−^stressed 3T3 fibroblast cells	Pleguezuelos-Villa et al. (2019)
		Reduced inflammatory infiltrates and MPO activity	
	Gambogic acid (**8**)	Inhibited *in vitro* keratinocyte proliferation of HaCaT cells	Wen et al. (2014)
		Inhibited activation of NF-κB by suppressing the TNF-α-induced translocation of p65 into nucleus	
		Downregulation of CD3^+^, IL-17 and IL-22	
		Decreased leukocyte recruitment and accumulation by downregulating ICAM-1, E-selectin, VEGFR-2 and p-VEGFR2	

IL, interleukin; TNF-α, tumor necrosis factor αXanthones and skin inflammatory diseasesXanthones and skin inflammatory diseases==========This is a provisional file, not the final typeset articl.

## Xanthones involved in the Treatment of Skin Inflammatory Diseases

### Atopic Dermatitis

AD, also known as atopic eczema, is the most common chronic skin disorder affecting 3-20% of the global population (Lopez Carrera et al., 2019; Schwingen et al., 2020). AD is commonly characterized by pruritic skin lesions with concurrent allergic inflammation and augmented immunoglobulin E (IgE) and histamine levels (Higuchi et al., 2013; Zhao et al., 2017). Allergic inflammation and augmented serum IgE levels underlie the immunological dysregulation and subsequent dysfunction of the skin barrier (Higuchi et al., 2013). Reactive oxygen species (ROS) have also been associated with the pathogenesis of skin allergic reactions (Bickers and Athar 2006; Sivaranjani et al., 2013). ROS causes lipid peroxidation and damages cellular components, initiating cell death (Okayama 2005; Sivaranjani et al., 2013). The generation of ROS also activates immune responses by inducing leukocyte infiltration and altering cytokine expression profiles of Th1 and Th2 cells (Bickers and Athar 2006). Thus, antioxidants and anti-inflammatory agents such as xanthones are highly beneficial to treat AD (Okayama 2005; Sivaranjani et al., 2013).

#### Anti-itching Activity & Histamine Modulation by Xanthones

Xanthones were found to attenuate various clinical symptoms of AD such as skin lesions and possess anti-itching effect. The xanthone-rich mangosteen rind of *G. mangostana* L. extracted with 70% ethanol was studied by Higuchi et al. (2013) as a potential preventative agent for AD. The extract was administered orally at a dose of 250 mg/kg/day to 6-week-old NC/Tnd mice which decreased the frequency and duration of scratching per 20 minutes in treated-NC-Tnd mice. This finding was linked to the decreased keratinocyte and fibroblast production of nerve growth factors (NGF) and nerve extension that relieved the itching.

Histamines, also known as imidazolethylamine, play an important role in cutaneous inflammation and contribute to the characteristic redness, wheal, flare and pruritis of AD (Buddenkotte et al., 2010). It affects the integrity of the skin barrier and modulates inflammatory immune responses via the activation of eosinophils, mast cells and basophils (Buddenkotte et al., 2010; Ohsawa and Hirasawa 2014). Furthermore, this allergic mediator is capable of activating Th2 cells, subsequently increasing the production of Th2 cytokines such as IL-4, IL-5, and IL-13 (Ohsawa and Hirasawa, 2014). While the pruritic role of histamines in AD is still being debated, administration of antihistamines did help to mitigate itching (Ohsawa and Hirasawa, 2014). A study on the anti-inflammatory activity of the 40% ethanol extract of mangosteen hull (*G. mangostana* L.) revealed more than 80% inhibition of histamine release from IgE-sensitized RBL-2H3 basophil cells, besides inhibiting the synthesis of prostaglandin E_2_ (PGE2) at 300 μg/mL (Nakatani et al., 2002). The key compound contributing to the anti-inflammatory effect of the extract was suggested to be a xanthone named as α-mangostin (**2**). Meanwhile, **1** was revealed to be capable of reducing serum levels of histamine in 2,4-dinitrofluorobenzene (DNFB)-induced AD mice model with 0.001-1.000 mM in a dose-dependent manner (Aye et al., 2020). DNFB is a hapten which is topically applied to animal models to mimic the pathophysiology and skin inflammation of AD (Aye et al., 2020). Furthermore, **1** demonstrated significant reduction in skin lesions and hyperplasia in DNFB-induced mice at doses of 0.2, 2.0 and 20.0 mg/kg (Aye et al., 2020). *In vitro* experiments further highlighted the ability of **1** at 0.001-1.000 mM to suppress histamine and thymic stromal lymphopoietin (TSLP) released by HMC-1 mast cells that was stimulated by phorbol myristate acetate (PMA) and calcium ionophore A23187 (CI). The synergistic combination of PMA-CI was established as powerful tools to study accessory-independent activation of T cells (Heinze and Mohr, 1990).

α-Mangostin (**2**) was previously reported to be a selective and competitive inhibitor of histamine H1 receptors (Chairungsrilerd et al., 1996a; Chairungsrilerd et al., 1996b). The antihistamine effect of **2** via the inhibition of histamine release and antagonism of histamine H1 receptors disclosed the xanthone as a potential candidate to alleviate histamine-mediated effects in allergic reactions such as AD. Besides **2**, γ-mangostin (**3**) is another major xanthone found in mangosteen rind and hull extracts (Nakatani et al., 2002; Higuchi et al., 2013). As shown in Figure 2, both **2** and **3** are similar in structures and only differ by demethylation of 7-OH in **2**. An early histamine-induced contraction study on **3** showed that it lacked of histamine H1 receptor antagonism on the isolated rabbit aorta at 5 μM (Chairungsrilerd et al., 1996b). However, a later study on neuroblastoma cells by Sukma et al. (2011) contradicted the findings by Chairungsrilerd et al. (1996b). The authors suggested that the increase in mRNA expression of H1 receptors is compensatory against the antagonistic effect of **3** (Sukma et al., 2011). Further investigation on the structure-activity relationship of xanthone on histamine antagonism may be worthwhile.

#### Modulation of Cytokine Expression and IgE by Xanthones

Xanthones are involved in the modulation of immune inflammatory responses and the expression of cytokines and IgE. The pristine xanthone **1** was found to reduce the mRNA expression of immunomodulatory mediators and ILs responsible for recruiting immune cells to the site of skin inflammation in mice at doses of 0.2, 2.0 and 20.0 mg/kg (Aye et al., 2020). The mediators involved are IL-1β, IL-6, IL-8, thymus and activation-regulated chemokine (TARC) and macrophage derived chemokine (MDC) (Aye et al., 2020). Xanthone **1** also reduced the serum levels of IgE, as well as the population of mast cells in skin lesions at the same doses. Furthermore, **1** increased the survival rate of anaphylactic shock by 83.3%, further highlighting its immunomodulatory capabilities. It is worthwhile to note that **1** did not induce any cytotoxicity to HaCaT normal keratinocyte cells at 0.001-10.000 mM (Aye et al., 2020). Another study on the xanthone-rich mangosteen rind ethanol extract using AD mice models revealed the suppression of mRNA expression of various pro-inflammatory cytokines such as IL-4, interferon-γ (IFN-γ) and TSLP when treated with 250 mg/kg/day of the extract (Higuchi et al., 2013). TSLP was suggested to be the most crucial target of the extract against AD development. Besides that, the extract at the same dose suppressed the mRNA expression of Th2 chemokines MDC and eotaxin-2 involved in T cell-mediated inflammation and recruitment of eosinophils, respectively (Watanabe et al., 2002; Higuchi et al., 2013). However, the expression of pro-inflammatory cytokines commonly associated with AD such as IL-4, IL-5 and IL-13 were not affected by treatment with the extract (Higuchi et al., 2013). Xanthone **2** was identified as the most abundant xanthone and main active compound of the mangosteen rind ethanol extract (Higuchi et al., 2013). The isolated **2** was further reported to inhibit the mRNA expression of TSLP and IFN-γ with IC_50_ values of 0.4 and 3.6 μM, respectively.

In contrast, mangiferin (**4**), a natural xanthone glucoside, was able to alleviate the imbalanced expression of cytokines in the serum of oxazolone-induced AD in C57BL/6 mice model with oral administration of 50 mg/kg/day (Zhao et al., 2017). Treatment with **4** restored the Th1/Th2 cytokine imbalance by reducing serum levels of pro-inflammatory cytokines including IL-4, IL-5, IL-13, IL-17A and TNF-α, while increasing the levels of anti-inflammatory cytokines IL-10 and transforming growth factor β (TGF-β) (Guo et al., 2014; Zhao et al., 2017; Yun et al., 2019). Besides that, 20 and 100 μM of **4** were shown to modulate cytokine expression of macrophages which play a crucial role in skin inflammation and reduce the levels of IL-1β, IL-6 and inducible nitric oxide synthase (iNOS) secreted by RAW264.7 cells (Zhao et al., 2017). Interestingly, **4** also directly affected the T cell population, particularly Th1, Th2, Th9, Th17 and Treg cells whereby imbalance of these subsets characterizes the impaired immune response in AD (Auriemma et al., 2013). Compound **4** can restore the aberrant Th1/Th2 cell ratio found in allergic inflammatory disorders while decreasing Th9 and Th17 proportions and increasing proportions of Treg cells in the spleen with oral administration of 50-200 mg/kg/day (Guo et al., 2014; Yun et al., 2019). The modulation of the Th1/Th2 cytokine and cell differentiation imbalance by **4** may involve the inhibition of STAT6 signaling pathway (Guo et al., 2014). Collectively, these anti-inflammatory mechanisms exhibited by **4** may contribute to the alleviation of skin inflammation in AD mice model (Zhao et al., 2017).

Besides that, **2** suppressed the IgE production induced by IL-4 and lipopolysaccharide (LPS)-stimulated splenic B cells with an IC_50_ value of 2.5 μM whereas a xanthone of similar structure, **3** showed an IC_50_ value of 1.6 μM (Higuchi et al., 2013). This finding was also previously described by Jang et al. (2012) where both **2** and **3** at the doses of 10 and 30 mg/kg/day were able to decrease the secretion of IgE in an airway inflammation model induced by ovalbumin, an allergen commonly used in immunological studies to imitate allergic reactions. Furthermore, the authors suggested the ability of **2** and **3** to inhibit allergen-IgE-induced mast cell activation while attenuating the production of IgE, therefore exhibiting anti-inflammatory effects through multiple mechanisms.

#### Other Anti-inflammatory Mechanisms of Xanthones

Besides mediating cytokine expression, xanthones were found to modulate the recruitment of eosinophils. Since eosinophils play a key role in allergic diseases, minimal recruitment of the cell consequently reduces the associated inflammation (Guo et al., 2014). Mangosteen rind ethanol extracts that primarily contain **2** and **3** reduced the population of eosinophils in the skin of NC/Tnd mice at a dose of 250 mg/kg/day (Higuchi et al., 2013). The finding may be associated with the ability of **2** and **3** to suppress PI3K activity, a signal transduction pathway commonly involved in the recruitment and activation of eosinophils, at a dose of 30 mg/kg/day (Jang et al., 2012). Similarly, **4** was able to dose-dependently suppress eosinophilia with comparable effects with dexamethasone (1-dehydro-9α-fluoro-16α-methylhydrocortisone), a topical corticosteroid used in the treatment of AD (Kalz 1960; Guo et al., 2014; Yun et al., 2019). This finding was consolidated by histological examination of the skin of oxazolone-induced AD in C57BL/6 mice that revealed a decrease in inflammatory cell infiltration after oral administration of **4** at 50 mg/kg/day (Zhao et al., 2017).

The inflammatory NF-κB pathway has been associated with AD. This pathway mainly involves the MAPK/NF-κB cascade and NF-κB/caspase-1 axis which induce the expression of numerous pro-inflammatory cytokines such as TNF-α, IL-8 and IL-1β (Lee et al., 2015; Guo et al., 2019). Modulation of the NF-κB pathway appears to be a common anti-inflammatory mechanism exhibited by xanthones. Xanthone **1** significantly attenuated the phosphorylation of IκBα and NF-κB in HaCaT cells at 0.001 to 10.000 mM, demonstrating the inhibition of an allergic inflammatory response (Aye et al., 2020). Besides that, **1** was also shown to regulate the MAPK and caspase-1 pathways in keratinocytes and mast cells (Aye et al., 2020). Its derivatives such as **2** was reported to inhibit the NF-κB pathway at 10 and 30 mg/kg/day in ovalbumin-induced airway inflammation in mice models, resulting in anti-inflammatory action (Jang et al., 2012). Compound **4** also suppressed the activation of the NF-κB pathway *in vivo* when oxazolone-induced dermatitis mice were treated with 50 mg/kg/day of the xanthone by reducing the level of NF-κB2 and inhibiting the phosphorylation of IκB. Thus, **4** was proposed to be a good lead compound for treating AD (Zhao et al., 2017). Similarly, xanthones **1** and **2** are potential lead compounds for treating AD as they exhibit anti-inflammatory effects.

### Acne

Acne vulgaris, a cutaneous inflammatory disease of the pilosebaceous follicle is listed as the eighth most prevalent disease worldwide and is estimated to affect 9.38% of the global population (Heng and Chew, 2020). Commensal skin bacteria *Propionibacterium acnes* and *Staphylococcus epidermidis* are associated with the onset and development of inflammatory acne via production of neutrophil chemotactic factors as well as pro-inflammatory cytokines such as IL-1, IL-6, IL-8 and TNF-α (Chomnawang et al., 2005; Asasutjarit et al., 2013; Xu et al., 2018). Recruitment of immune cells subsequently induced the generation of ROS which contributes to the irritation of the follicular epithelium that often leads to skin damage and scarring (Chomnawang et al., 2007). These acne-causing bacteria are quickly gaining resistance to classic antibiotics, hence the search for natural antibacterial and anti-acne compounds is ongoing (Asasutjarit et al., 2013). Xanthones are compounds that can be potentially used in treating acne.

#### Anti-acne Activity of Xanthone-rich Extracts

*Garcinia mangostana* L. ethanol extracts and its primary xanthone **2** were proven to exhibit anti-acne effects (Chomnawang et al., 2007; Pan-In et al., 2015). Early studies reported that the xanthone-rich ethanol extract showed the strongest inhibitory effects with minimum inhibitory concentration (MIC) of 0.039 mg/mL on both *P. acnes* and *S. epidermidis* when compared to eighteen Thai medicinal plant extracts with MICs ranging from 0.625 to more than 5 mg/mL using disc diffusion and broth dilution methods (Chomnawang et al., 2005). The extracts were analyzed using bioautographic detection in thin layer chromatography, and silica gel column chromatography which identified the main components of the extract to be **2** and **3** (Chomnawang et al., 2005; Tatiya-Aphiradee et al., 2016). Another study demonstrated the anti-microbial effects of aqueous extracts of *G. mangostana* L. on both acne-causing bacteria using a similar disc diffusion assay, further supporting Chomnawang and colleagues’ discoveries (Bhaskar et al., 2009). Later studies reported that the dichloromethane extract of mangosteen fruit rind (*G. mangostana* L.) obtained using Soxhlet extraction showed the strongest bacteriostatic and bactericidal activity against *P. acnes* and *S. epidermidis* with minimal bactericidal concentration (MBC) of 3.91 and 15.63 μg/mL, respectively (Pothitirat et al., 2010). Similarly, **2** was isolated and identified to be the key active compound in the rind extract. The concentration of **2** in the extract was significantly correlated to antibacterial activity against *P. acnes* and *S. epidermidis* (Pothitirat et al., 2010). Meanwhile, in a 3-week randomized, double-blinded and placebo-controlled study, mangosteen rind extract capsules were orally prescribed thrice daily to 94 patients with mild and moderate acne (Sutono 2013). The study reported a decrease of 63% and 35% in non-inflamed and inflamed acne lesions respectively, with no adverse effects (Sutono 2013). It was concluded that oral administration of the rind extract significantly decreased the severity of mild and moderate acne compared to a placebo.

#### Antibacterial Activity of Xanthones for Acne Treatment

Further investigations on isolated xanthone **2** from *G. mangostana* L. pericarp ethanol extract reveal its bacteriostatic (Tatiya-Aphiradee et al., 2016) and bactericidal effects (Koh et al., 2013) on Gram-positive bacteria using broth dilution and time-kill kinetics assays. Koh et al. (2013) identified the isoprenyl group attached at C-8 of **2** to be crucial in its antibacterial effect as it allowed the xanthone to associate with bacterial lipid alkyl chains and penetrate into the bacteria. Furthermore, a separate study highlighted that **2** mimics the chemical structure of *meta*-phenylene ethynylene, a membrane-targeting cationic antimicrobial peptide (Sivaranjani et al., 2017). Consequently, rapid penetration of the xanthone into the bacterium deranged the cytoplasmic membrane integrity, causing swift loss of intracellular components within 5-10 minutes at concentrations as low as 7.61 μM as proven in an ethidium bromide uptake assay, together with SYTOX green and calcein leakage assays (Koh et al., 2013). Thus, it was postulated that the primary target of **2** in antibacterial mechanisms of Gram-positive bacteria, such as *P. acnes* and *S. epidermidis* is the bacterial inner membrane. This discovery was also corroborated by Auranwiwat and colleagues (2014) that found the prenyl substituent at C-8 in α-mangostin (**2**), β-mangostin (**5**) and garcicowanone A (**6**), a novel xanthone identified in the unripe fruits of *Garcinia cowa* Roxb. ex Choisy, to confer to their remarkable antimicrobial activity against Gram-positive bacteria. Xanthones **2**, **5** and **6** are similar in structure, differing by prenyl substituents at C-2, and hydroxyl or methoxy groups at C-3 positions as shown in Figure 2. Moreover, **2**, **5** and **6** demonstrated significant activity against *S. epidermidis* with MIC values between 2-4 μg/mL, where **2** is the most potent among the xanthones (Auranwiwat et al., 2014). Hence, it was postulated that prenyl and hydroxyl groups attached at C-2 and C-3 positions, respectively of **2** were the important functional groups for antibacterial activity. Additionally, **2** significantly inhibited biofilm formation of *S. epidermidis*, a crucial virulence factor of the bacterium at concentrations as low as 12.18 μM (Sivaranjani et al., 2017). Another study reported the anti-*P. acnes* activity of **2** and **3** (Xu et al., 2018). Interestingly, while **2** and **3** displayed similar MIC values of 4.0 μM on *P. acnes* growth, **3** achieved a larger maximum inhibition zone of 20 mm diameter compared to 12 mm diameter by **2** in the disc diffusion method. This finding suggests **3** to be a more potent anti-bacterial agent against *P. acnes* compared to **2** though there is still a lack of research study on the anti-acne activity of **3** (Xu et al., 2018). Thus far, no comparative studies have been done on the anti-acne activity of **2**, **3** and **5** which have closely related structures with prenyl substituent at C-2 and C-8.

Previous studies reported that **2** is effective against methicillin-resistant *Staphylococcus aureus* (MRSA) strains in an *in vivo* model by using superficial skin infection mouse model (Tatiya-Aphiradee et al., 2016). Topical treatment with 100 μL of 1.32% of **2** significantly reduced the number of MRSA colonies after 3 days to levels comparable to the control (Tatiya-Aphiradee et al., 2016). Interestingly, studies to date reported no resistance development towards **2** in MRSA with insignificant changes in the MIC of 3.80 μM when tested in a 20-passage multistep resistance selection study (Koh et al., 2013). Similarly, *S. epidermidis* did not develop resistance towards **2** despite continuous exposure at 0.38-3.05 μM across 19 passages in both planktonic and biofilm cells (Sivaranjani et al., 2017). A later study conducted by Sivaranjani et al. (2019) revealed molecular antibacterial mechanisms of **2** on *S. epidermidis* by downregulating the expression of genes responsible for bacterial cytoplasmic membrane integrity, cell division, teichoic acid and fatty acid biosynthesis, as well as DNA replication and repair systems. The target on multiple metabolic pathways by **2** minimized the risk of resistance development which greatly increased the potential of the xanthone as anti-bacterial agents for acne treatment.

#### Anti-inflammatory Mechanisms of Xanthones

Investigations on the potential of *G. mangostana* L. pericarps ethanol extracts for acne treatment revealed its anti-inflammatory effects by suppressing the production of pro-inflammatory cytokine, TNF-α. The pericarp extract suppressed the production of TNF-α by 94.59% at 50 μg/mL in an *in vitro* cytokine production assay in peripheral blood mononuclear cells stimulated with heat-killed *P. acnes*. (Chomnawang et al., 2007). The extract also possessed antioxidative activity by free radical scavenging mechanisms as shown in DPPH scavenging assay (IC_50_ = 6.13 μg/mL). The authors suggested the potential anti-inflammatory mechanisms was due to the attenuation of oxidative stress. A recent study revealed **2** and **3** to exhibit anti-acne activities via multiple anti-inflammatory mechanisms (Xu et al., 2018). The mRNA expression of pro-inflammatory cytokines IL-1β, IL-6 and TNF-α in *P. acnes*-stimulated HaCaT cells was significantly inhibited by **2** and **3** at concentration of 2-8 μM, as shown in a qRT-PCR analysis (Xu et al., 2018). Furthermore, **2** and **3** also suppressed the activation of NF-κB and MAPK pathways by decreasing the phosphorylation of pathway-related proteins IκB, p65, p38, ERK and JNK in a dose-dependent manner (Xu et al., 2018). Moreover, these xanthones completely inhibited the activity of lipases, a pro-inflammatory and virulence factor of *P. acnes*, at concentrations as low as 4 μM. Consequently, **2** and **3** halted the inflammatory response induced by *P. acnes* (Xu et al., 2018). Various anti-inflammatory mechanisms exhibited by these xanthones contribute to their biological potential as anti-acne agents.

#### Development of Xanthone Formulations for Acne Treatment

As a hydrophobic prenylated xanthone, flux of **2** through the stratum corneum is undesirable for acne treatment as it limits drug retention and absorption in hair follicles and sebaceous glands (Bhaskar et al., 2009; Pan-In et al., 2015). Numerous topical formulations were hence designed to maximize the retention of active compounds within the epidermis, ideally attaching to the bacterial surface and directly releasing the xanthone to the bacteria (Bhaskar et al., 2009; Pan-In et al., 2015). For example, an anti-acne gel formulation in aqueous based Carbopol-934 (1% w/w) containing aqueous extracts of *G. mangostana* L. and *Aloe vera* (L.) Burm.f. was developed for the topical therapy of mild acne (Bhaskar et al., 2009). The formulation had an ideal hydrophilic-lipophilic balance for epidermal permeation and showed larger maximum zone of inhibition of 1.7 cm against *P. acnes* compared to the marketed clindamycin phosphate gel of 1.1 cm when 1.5 g of the gels were tested using a zone of inhibition assay (Bhaskar et al., 2009). Interestingly, the formulated gel was claimed to be advantageous as there was no development of tolerance or resistance by the bacteria. Identification of **2** as the crucial phytochemical in these mangosteen extracts led to the anti-acne studies involving the film-forming solutions of a purified α-mangostin (**2**)-rich mangosteen fruit rind extract (*G. mangostana* L.) (Asasutjarit et al., 2013). The optimized formulations utilized 8% w/w of Klucel LF and Eudragit RL PO in a 5:1 ratio as film-forming polymers and 1% w/w triethyl citrate as a plasticizer. With 1 mg/g of **2** loaded, the films showed significant anti-bacterial activity against *P. acnes* in the disc diffusion method, producing a inhibition zone diameter of 23.5±1.5 mm. Similarly, nanoemulsions of **2** showed anti-bacterial activity against *P. acnes* while also being non-toxic to HaCaT cells at concentration of 0.2% w/w of **2** in nanoemulsions (Asasutjarit et al., 2019). In addition, other studies employed cellulose-based nanoparticles for delivery of **2** and showed insignificant skin irritation *in vivo* on human volunteers in a 4-week-randomized, double-blind, placebo-controlled, split-face study with topical application of 0.3, 0.6 and 1.2% w/w **2**-loaded nanoparticles (Pan-In et al., 2015). The non-irritancy may be associated with the non-cytotoxicity of **2** towards normal keratinocytes and fibroblasts. For example, an *in vitro* examination showed the formulation of the xanthone-rich extract to be non-toxic to normal skin fibroblast cells and was regarded to be non-irritant and safe for skin use (Asasutjarit et al., 2013). This finding was also supported by Xu et al. (2018) that showed **2** and **3**, the major xanthones of mangosteen pericarps (*G. mangostana* L.), to be non-toxic to HaCaT keratinocyte cells despite showing effective antibacterial activity against *P. acnes*. Moreover, these xanthones inhibited *P. acnes*-induced hyperproliferation of HaCaT cells at 2-8 μM in a dose-dependent manner. There was no significant effect on the proliferation of non-induced HaCaT cells at the same concentrations which excluded the non-specific cytotoxicity of the xanthones as a mechanism of action.

### Skin Cancer & Melanoma

Skin cancer is classified as either melanoma or non-melanoma where melanoma is regarded as the most aggressive form of skin cancer (Delgado-Hernández et al., 2019). Non-melanoma skin cancer is also ranked as the fifth most common cancer worldwide by the World Health Organization (WHO) (Khazaei et al., 2019). Meanwhile, the incidence of melanoma is increasing at a faster rate than any other cancer with worrying mortality rates (Purnaningsih et al., 2018; Khazaei et al., 2019). UV radiation in particular has been strongly associated with skin carcinogenesis, causing oxidative damage, pro-inflammatory responses and DNA mutations (Hiraku et al., 2007; Kapadia et al., 2013; Im et al., 2017). Inflammation was described to be crucial in this skin disease as the inflammatory environment supports tumorigenesis and promotes invasion and metastasis (Pittayapruek et al., 2016; Wang et al., 2017). The extent of inflammation is commonly assessed by the detection of pro-inflammatory cytokines such as TNF-α, IL-1β, IL-4, IL-6 and IL-18 (Wang et al., 2017). ROS was also implicated in the initiation, promotion and progression stages of skin carcinogenesis by causing structural alterations in DNA and proteins (Bickers and Athar, 2006). Accumulation of ROS triggers inflammation and the production of matrix metalloproteinases (MMPs) that promote tumor invasion, metastasis and angiogenesis by mediating TGF-β and vascular endothelial growth factor (VEGF) (Pittayapruek et al., 2016; Im et al., 2017). The mechanism of action of xanthones commonly involve the apoptotic Bax and anti-apoptotic Bcl-2 proteins which are two of the most important markers of apoptosis (Wang et al., 2017). Activation of Bax and inhibition of Bcl-2 induce mitochondrial outer membrane permeabilization, apoptosome formation and activation of the executioner caspases-3 and -7 (Campbell and Tait, 2018). Consequently, the cell begins to dismantle, and apoptosis occurs. Other mechanisms of action include cell cycle arrest, and the disruption of MAPK and PI3K/Akt signaling pathways which modulate the growth and proliferation of skin cancer cells (Hambright et al., 2015).

There is a lack of a reliable therapy for this skin malignancy (Delgado-Hernández et al., 2019). Nowadays, multitargeted treatment is urgently required as singularly targeting a kinase or pathway is ineffective to cease malignant proliferation, angiogenesis, and metastasis. Therefore, xanthones with its multitargeting mechanisms are promising pharmacophore candidates for cancer treatment (Delgado-Hernández et al., 2019).

#### Cytotoxic Mechanisms of Xanthones

The cytotoxic mechanisms of various natural xanthones have been thoroughly investigated. The natural prenylated xanthone, **2** inhibited the *in vitro* proliferation, adhesion, and invasion of several melanoma cell lines, namely SK-MEL-28, B16-F10 and A375 in a dose-dependent manner (Wang et al., 2011; Beninati et al., 2014). This xanthone also induced apoptosis in melanoma SK-MEL-28 cells whereby it increased the proportion of early apoptotic cells from 1.7% (control) to 59.6% at concentration of 24.36 μM (Wang et al., 2011). The observed apoptotic effect was linked to the 25-fold increase in caspase-3 activity and loss of mitochondrial membrane potential (Δ*Ψ*_*m*_) (Wang et al., 2011). This finding was supported by Wang et al. (2017) who reported an increased expression of pro-apoptotic proteins, Bax, BAD and caspase-3 with concurrent downregulation of anti-apoptotic proteins, Bcl-2 and Bal-xL after *in vivo* treatment of mice with 5 and 20 mg/kg/day of **2**. Furthermore, the study by Wang and colleagues (2017) showed that **2** was active *in vivo* as it inhibited tumor incidence and hyperplasia induced by carcinogen 9,10-dimethylbenz[a]anthracene (DMBA) and skin tumor promoter 12-*O*-tetra-decanoylphorbol-13-acetate (TPA) in ICR mice following daily intraperitoneal administration of 5 mg/kg or 20 mg/kg of the xanthone (Wang et al., 2017). The inhibition of cancer cell proliferation was hypothesized to be linked to the suppression of the PI3K/Akt signaling pathway by decreasing the phosphorylation of PI3K, Akt and TOR proteins (Wang et al., 2017; Xia and Sun 2018). The use of 5 μM of **2** as an adjunct to kinase inhibitors of the PI3K pathway such as rapamycin was also shown to be effective in inhibiting the proliferation of SK-MEL-28 cells (Xia and Sun 2018). Interestingly, despite antioxidative and ROS scavenging activity of **2** (Pedraza-Chaverri et al., 2009; Pérez-Rojas et al., 2009), the induction of ROS generation in melanoma cells may be a cytotoxic mechanism exhibited by the synergistic combination of **2** and kinase inhibitors (Xia and Sun 2018). The structurally similar xanthone, **3** and another prenylated xanthone, 8-deoxygartanin (**7**) also exhibited similar inhibitory effects on SK-MEL-28 cell proliferation while increasing apoptotic rate and loss of Δ*Ψ*_*m*_ when tested at 2.5, 5.0 and 10.0 μg/mL (Wang et al., 2011). Among these three xanthones, **2** was the most potent as it exhibited the highest apoptotic and anti-proliferative activity against SK-MEL-28 cells.

The anti-metastatic effect of **2** was also reported where it induced cancer cell differentiation after treatment with 10 and 15 μM of the xanthone as evidenced by the intracellular increase of the differentiation marker PpIX in B16-F10 cells (Beninati et al., 2014). Furthermore, **2** reduced the plasticity of melanoma cells by up to 80% as seen in 3D-invasion assays (Beninati et al., 2014). The activity of MMP-9 which promotes angiogenesis and tumor invasion was reduced by 63% whereas cell aggregation increased by 3-fold when treated with 15 μM of **2**. Similarly, later studies conducted by Im et al. (2017) reported a reduction in MMP-1 and MMP-9 enzyme levels in the skin of UVB-irradiated mice after treatment with 100 mg/kg/day of **2**. These MMPs mediate the degradation of extracellular matrix which allow cancer cells to detach from the original tumor site and spread to other locations (Pittayapruek et al., 2016). Numerous mechanisms exhibited by **2** result in the anti-metastatic effect of xanthones.

Another xanthone with significant anti-cancer activity found in the resins of *Garcinia hanburyi* Hook.f. is gambogic acid (**8**) (Zhao et al., 2008). This caged xanthone exerted *in vitro* cytotoxicity on B16-F10 (IC_50_ = 1.71 μM) and A375 melanoma cells (IC_50_ = 2.50 μM), inhibiting the viability and cell proliferation in a dose-dependent manner (Zhao et al., 2008; Xu et al., 2009). Various mechanisms such as cell cycle arrest, release of cytochrome c from mitochondria as well as the induction of apoptosis and necrosis have been attributed to the cytotoxic effects of **8** (Zhao et al., 2008; Xu et al., 2009). For instance, treatment with 3.96-11.89 μM of **8** increased the number of early apoptotic A375 cells by at least 7-fold after 36 hours where the observed finding was associated with an increase in Bax, a decrease in Bcl-2, and subsequent increased activation of caspase-3 (Xu et al., 2009). Interestingly, co-treatment of **8** with cisplatin, a standard chemotherapy drug significantly increased cisplatin-induced cytotoxicity on cisplatin-resistant A375 melanoma cells, suggesting the potential of xanthone in combination therapy (Liang et al., 2018). Later studies reported the involvement of other apoptotic pathways in melanoma cells such as mitochondrial p66^shc^/ROS-p53/Bax (Liang and Zhang 2016) and a novel miR-199a-3p/ZEB1 signaling (Liang et al., 2018) in the anti-tumour mechanism of **8**. Besides that, **8** inhibited the migration and adhesion of B16-F10 melanoma cells to fibronectin via the downregulation of the cell adhesion molecule α_4_ integrin with 0.01-0.60 μM of **8** in a dose-dependent manner (Zhao et al., 2008). Moreover, lung metastases were significantly suppressed with an inhibitory rate of 94.14% by 1.5 mg/kg of **8** in an artificial *in vivo* metastasis assay in mice (Zhao et al., 2008). The anti-proliferative effect on the *in vivo* tumors was also associated with the decreased expressions of Akt1 and Akt2, proteins of the PI3K/Akt pathway which regulate cell proliferation (Hambright et al., 2015; Liang et al., 2018).

Similarly, another caged xanthone, gambogenic acid (**9**) induced apoptosis in B16 cells via the PI3K/Akt/mTOR signaling pathway (Cheng et al., 2014). This xanthone decreased phosphorylated levels of PI3K, Akt and mTOR in a time-dependent manner, limiting the activation of this proliferation-inducing pathway (Cheng et al., 2014). Cytotoxicity evaluation of the compounds isolated from *G. hanburyi* Hook.f. revealed two derivatives of **8**, 33-hydroxyepigambogic acid (**10**) and 35-hydroxyepigambogic acid (**11**) to have potent activity against SK-MEL-28 cells with IC_50_ values of 0.82 and 0.73 μM, respectively (Xu et al., 2015). Similar to **8**, these two caged xanthones instigated cell cycle arrest in S or G2/M phases and induced apoptosis through amplification of caspase-3/7 activity (Xu et al., 2015). Likewise, xanthone V_1_ (**12**), a compound isolated from a Cameroonian medicinal plant, *Vismia laurentii* De Wild. caused cell cycle arrest at the S phase and apoptosis via caspase-3/7 activation (Kuete et al., 2011). Interestingly, previous studies highlighted the apparent selectivity of **2**, **3** and **7** for cancer cells with higher IC_50_ values for CCD-1064Sk, a normal skin fibroblast cell line of 17.71, 28.58 and 27.21 μM, respectively when compared to SK-MEL-28 cells with IC_50_ of 14.42, 8.95 and 10.07 μM, respectively (Wang et al., 2011). Other compounds such as **12** was also found to be less toxic to AML12 normal hepatic cells (IC_50_ > 0.051 μM) compared to Colo-38 melanoma cells (IC_50_ = 0.003 μM) (Kuete et al., 2011).

Meanwhile, the glycosylated xanthone **4** did not exhibit significant cytotoxic effects on B16-F10 melanoma and EA.hy926 endothelial cells (Delgado-Hernández et al., 2019). Instead, this xanthone dose-dependently suppressed basic fibroblast growth factor (bFGF)-induced cell motility, metastasis, and angiogenesis at 30-120 μM as shown in an *in vitro* wound healing model, as well as chorioallantoic membrane and endothelial fibrin gel sandwich assays. Furthermore, genes such as VEGFR2, MMP-19 and PGF that contribute to cancer angiogenesis and metastasis processes were selectively inhibited by **4**, consequently ceasing cell invasion and migration (Delgado-Hernández et al., 2019). Meanwhile, its metabolite norathyriol (**13**) was found to inhibit cell proliferation of JB6 P+ mouse skin epidermal cells at 10 and 25 μM by causing cell arrest at the G2/M phase (Li et al., 2012). Furthermore, *in vivo* experiments showed the inhibition of skin carcinogenesis in SKH-1 hairless mice upon topical application of 0.5 or 1.0 mg of **13** in 200 μL of acetone before solar UV irradiation. These findings suggested **13** that possesses chemopreventive effects (Li et al., 2012).

#### Anti-inflammatory Mechanisms of Xanthones

Anti-inflammatory activity is a potential mechanism to treat skin cancers (Rayburn et al., 2009). The anti-inflammatory activity of **2** on immune cells RAW264.7 and THP-1, as well as various cancer cell lines such as HepG2, Caco-2 and HT-29 have been previously investigated (Gutierrez-Orozco et al., 2013; Mohan et al., 2018). A recent study reported the ability of 5 and 20 mg/kg/day of **2** to suppress the inflammation caused by DMBA-TPA-induced skin tumorigenesis in mice by downregulating skin and systemic levels of pro-inflammatory cytokines IL-1β, IL-4 and IL-18, and upregulating anti-inflammatory cytokine IL-10 (Wang et al., 2017). This xanthone also downregulated the inflammatory IL-1β, IL-6 and TNF-α at a transcriptional level in UVB-exposed hairless mice with oral administration of 100 mg/kg/day of **2** (Im et al., 2017). Meanwhile, 240 μM of **4** was found to suppress lipid and calcium signaling as well as cancer-inflammation via selective inhibition of multiple pro-inflammatory NF-κB genes, including IL-6, TNF, IFN-γ and CCL2 genes (Delgado-Hernández et al., 2019). Furthermore, **4** was found to significantly increase the antioxidant superoxide dismutase (SOD) activity in the skin of UVB-irradiated SKH-1 mice upon topical application of 100 μL of 4 mg/mL of **4** in ethanol:acetone (1:1, v/v). However, the activity and expression of antioxidant enzymes catalase (CAT) and cyclooxygenase (COX)-2 were not affected (Petrova et al., 2011). Nevertheless, **4** was capable of inhibiting UVB-induced edema in SKH-1 mice by 73.33% with topical application of 4 mg/mL of the xanthone (Petrova et al., 2011).

Norathyriol (**13**) showed anti-inflammatory activity *in vitro* via competitive inhibition of MAPK1 at its ATP-binding site at 10 and 100 μM (Li et al., 2012). The xanthone moiety acts as an adenine mimic, forming hydrogen bonds and hydrophobic interactions in the hinge region of MAPK1. Consequently, MAPK cascades as well as the UVB-induced activation and activity of pro-inflammatory transcription factors AP-1 and NF-κB were halted (Li et al., 2012). A recent fascinating study by Silva and colleagues (2019) investigated the modulatory activity of synthetic 1,2-dihydroxyxanthone (**14**) on THP-1 macrophage activity and A375-C5 melanoma cells (Silva et al., 2019). Treatment with 50 and 100 μM of **14** suppressed the expression of IL-1β and IL-10 by THP-1 macrophages. However, the production of TNF-α was found to increase, suggesting favorability for melanoma treatment based on a previous meta-analysis that linked the development of skin cancers to the use of TNF inhibitors (Mariette et al., 2011; Silva et al., 2019). However, this finding highlighted the contradictions in current literature on the role of TNF-α in skin cancer and melanoma whereby other studies did not support the benefits of upregulating the cytokine (Smith et al., 2014; Mercer et al., 2017). Further studies on the immune modulation and underlying mechanisms in skin cancers should hence be considered.

#### Photoprotective Activity of Xanthones for Chemoprevention

While anti-inflammatory action potentially attenuates cancer development and progression, the anti-inflammatory effect of xanthones may also protect normal tissues from cellular damage (Rayburn et al., 2009). Hence, xanthones were highlighted to be photoprotective and chemopreventive agents. A recent study by Purnaningsih et al. (2018) demonstrated the chemopreventive activity of topical applications of 100, 200 and 400 ppm of mangosteen skin (*G. mangostana* L.) ethanol extracts which predominantly consists of **2**, **3** and **5** using a DMBA-induced mice skin cancer model. The authors hypothesized that the observed chemopreventive effect of the extract came from the antioxidant activity and attenuation of ROS production by **2**, **3** and **5** (Purnaningsih et al., 2018). This hypothesis was also consolidated by previous studies that showed **2** isolated from *G. mangostana* L. pericarps to exhibit antioxidative activity by increasing the antioxidant activity of SOD and CAT, halting the generation of oxidative stress (Im et al., 2017).

Besides that, the ethanol and aqueous extracts of two species of honeybush, *Cyclopia intermedia* E.Mey. and *C. subternata* Vogel, which predominantly contain **4** were found to exert *in vitro* cytoprotective effects with negligible effects on the cell viability of normal HaCaT keratinocytes and CRL-7761 fibroblasts (Magcwebeba et al., 2016a). The lack of activity may be associated with poor solubility of the xanthone or influenced by the xanthone-flavanone ratio in the extract. Nonetheless, the study highlighted the high redox potential of **4** and its ability to counter oxidative stress via nuclear factor erythroid 2-related factor 2 (Nrf2) modulation which may additively or synergistically act with other polyphenolic constituents in the honeybush extract to protect against UVB irradiation (Magcwebeba et al., 2016a; Magcwebeba et al., 2016b). Another study highlighted the cytoprotective ability of **4** to inhibit UVB-induced edema and lipid peroxidation in an *in vivo* study (Petrova et al., 2011). Moreover, this xanthone reduced metal-induced oxidative stress and mitochondrial dysfunction via iron-chelating mechanisms in lipid membranes (Magcwebeba et al., 2016a). It was proposed that antioxidative compounds are able to relieve cancer-inflammatory damages as a result of oxidative stress and prolonged UV exposure (Kapadia et al., 2013). Therefore, **4** was suggested to be effective photoprotective agents with potential applications as sunscreens, cosmetics or skin care products to protect against UVB-induced oxidative stress and the resulting skin inflammation (Petrova et al., 2011; Magcwebeba et al., 2016b). Interestingly, two other xanthone derivatives gentiacaulein (**15**) and norswertianin (**16**) were identified to be chemopreventive agents that utilized a novel quenching mechanism instead of antioxidation or physical sunscreen effects (Hirakawa et al., 2005). Using fluorescence spectroscopic techniques and *ab initio* molecular orbital calculations, the DNA photoprotective effects of these xanthones were found to involve the quenching of the triplet photoexcited state of riboflavin (Hirakawa et al., 2005).

### Psoriasis

Psoriasis is a chronic T-cell mediated skin inflammatory disease characterized by thickened and scaly plaques and skin lesions (Wen et al., 2014; Owczarek and Jaworski, 2016). Pathogenesis of this disease involves interaction between genetic and environmental factors. Histological symptoms commonly involve keratinocyte hyperproliferation, abnormal keratinocyte differentiation and infiltration of inflammatory cells (Wen et al., 2014; Pleguezuelos-Villa et al., 2019). The production of inflammatory cytokines such as the Th17 signature IL-17A, IL-22, IL-23 and IFN-γ is a key characteristic of psoriasis (Schön, 2019). Dysregulated effector T-cell function and differentiation as well as the NF-κB pathway were also associated to the inflammatory response in psoriasis. Besides that, the influx of neutrophils in psoriatic lesions is a histological characteristic of psoriasis (Guérard et al., 2013). Neutrophils were reported to play a crucial role in psoriasis besides being a key source of the pro-inflammatory cytokines IL-8 and IL-17A (Biasi et al., 1998; Wang and Jin, 2020). Recently, the role of ROS and oxidative stress in psoriasis was demonstrated (Khmaladze et al., 2015; Pleguezuelos-Villa et al., 2019). The ROS generated by keratinocytes, fibroblasts and skin endothelial cells produces chemotactic effects towards neutrophils thereby increasing their infiltration into psoriasis lesions. The activated neutrophils generate a large amount of superoxide anion (O_2_^•−^) in a process known as respiratory or oxidative burst (Khmaladze et al., 2015). The interaction between psoriatic keratinocytes and neutrophils was reported to increase the lifespan of this immune cell and augment its production of O_2_^•−^, resulting in the oxidative stress (Guérard et al., 2013). Thus, it was suggested that the use of antioxidants are ideal to treat this inflammatory disorder (Pleguezuelos-Villa et al., 2019). As a rich source of natural antioxidants and anti-inflammatory compounds, all parts of the xanthone-rich *G. mangostana* L. plant were traditionally consumed and utilized for the treatment of psoriasis (Ibrahim et al., 2018a; Ibrahim et al., 2018b; Vemu et al., 2019).

#### Anti-inflammatory Mechanisms of Xanthones

Gambogic acid (**8**) was investigated for its potential use in psoriasis treatment. This compound can counter keratinocyte hyperproliferation, a hallmark of psoriasis as it was shown to be able to inhibit *in vitro* proliferation of normal HaCaT keratinocytes with an IC_50_ of 0.09 μM (Wen et al., 2014). Treatment with **8** also suppressed the translocation of p65 into the nucleus upon TNF-α induction and therefore prevented the subsequent activation of NF-κB and its inflammatory response. In addition, *in vivo* studies on the K14-VEGF transgenic mice model which develops a psoriasis-like cutaneous inflammation showed a dose- and time-dependent inhibition of epidermal hyperplasia, inflammatory infiltrates and hyperkeratosis with topical application of a cream containing 0.10, 0.25 or 0.50% of **8** (Wen et al., 2014). The anti-psoriatic effect of **8** was also observed in psoriasis-like guinea pig and mouse tail models with great improvement in morphological and histological examinations (Wen et al., 2014). In the same study, immunostaining of CD3^+^, IL-17 and IL-22 revealed downregulation of these inflammatory factors upon treatment with creams containing 0.10, 0.25 or 0.50% of **8**. Downregulation of cell adhesion molecules such as ICAM-1 and E-selectin which play a crucial role in leukocyte recruitment and accumulation in the psoriatic skin was also observed after the treatment. Furthermore, the expression and phosphorylation of VEGF signaling transducer, VEGFR-2 was downregulated by **8**, consequently suppressing VEGF-mediated angiogenesis and production of adhesion molecules during inflammation (Wen et al., 2014).

Studies on naturally derived xanthones for psoriasis treatment are still limited. As an inflammatory disease, treatment of psoriasis commonly involves anti-TNF agents (Zhao et al., 2017). Inhibitory effects towards TNF-α are commonly observed as exhibiting anti-inflammatory effects of xanthones including **2** (Gutierrez-Orozco et al., 2013; Lee et al., 2013; Im et al., 2017; Herrera-Aco et al., 2019), **3** (Xu et al., 2018; Chiu et al., 2020), **5** (Lee et al., 2013) and **8** (Wang et al., 2018b) are considered potential compounds for psoriasis treatment. A complication commonly associated with psoriasis is inflammatory arthritis where pathogenesis involves similar immunological aberrations of inflammatory cytokines such as IL-17, IL-22, IL-23 and IFN-γ (Sankowski et al., 2013; Schön, 2019). The inflammatory effects of xanthones against arthritis have been previously studied, hence further investigations for the application of these natural xanthones for psoriasis may be considered. One of these xanthones, **2** exhibited anti-inflammatory effects in collagen-induced arthritis in the classic arthritis DBA/1J mice with oral administration of 10 and 40 mg/kg/day via the reduction of pro-inflammatory cytokines IL-6 and IL-33, as well as chemokines CXCL5, CXCL10, CXCL9 and CCL5 which are responsible for the recruitment of inflammatory cells (Herrera-Aco et al., 2019). Similarly, inhibitory effects on the secretion of TNF-α and IL-6 were observed in LPS-stimulated mice with ED_50_ of less than 100 mg/kg (Lee et al., 2013). Besides that, **3** was able to relieve inflammatory symptoms of osteoarthritis by inhibiting the expression of pro-inflammatory transcription factors STAT3 and NF-κB, as well as the expression of cytokines IL-6 and TNF-α (Chiu et al., 2020).

Recent studies proposed that xanthones such as **4** have great potential in treating psoriasis due to its ability to inhibit the pro-inflammatory effects of TNF-α (Zhao et al., 2017). For instance, the anti-inflammatory effects of **4** was highlighted to suppress TNF-α, IL-1β and IL-6 expressions in collagen-induced inflammatory arthritis DBA/1J mice by attenuating the activation of the inflammatory NF-κB and MAPK pathways after oral administration of 100 and 400 mg/kg/day of the xanthone (Tsubaki et al., 2015; Delgado-Hernández et al., 2019). However, the applications of xanthones are usually limited by their low bioavailability (Pleguezuelos-Villa et al., 2019). Thus, a recent study developed biocompatible glycethosome vesicles to stably incorporate **4** and deliver it to the epidermis besides permitting drug accumulation in the skin. Since oxidative stress is involved in psoriasis pathogenesis, the antioxidant effects of glycethosome vesicles containing 2, 4, 6, and 8 mg/mL of **4** was investigated *in vitro* using H_2_O_2_-stressed 3T3 fibroblast cells and the results showed virtually 100% cytoprotective effects on the cells (Pleguezuelos-Villa et al., 2019). Furthermore, in TPA-induced mice models, topical application of the **4**-loaded vesicles reduced inflammatory infiltrates, myeloperoxidase (MPO) activity, edema as well as abnormal epidermal alterations (Pleguezuelos-Villa et al., 2019).

**FIGURE 3:**
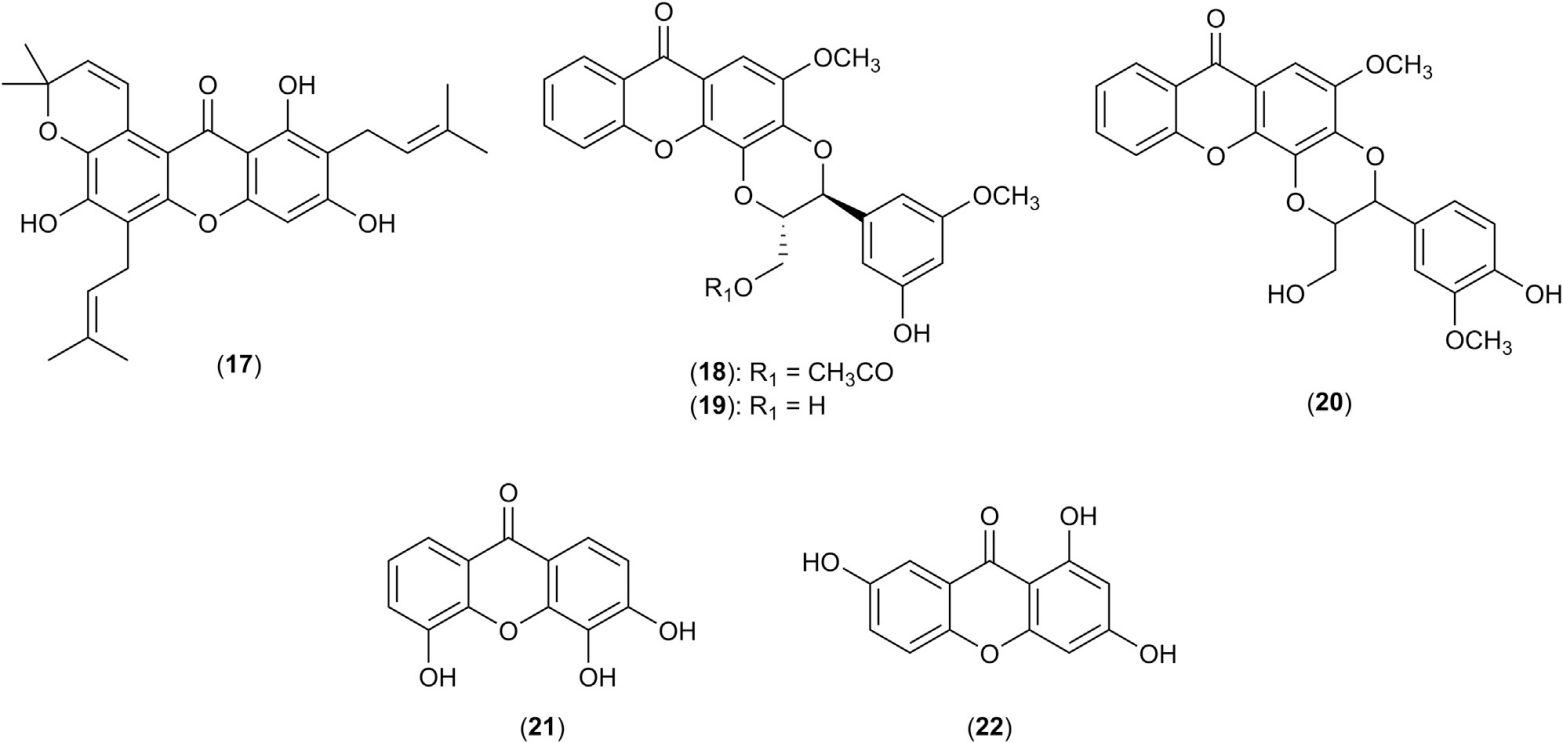
Structures of tovophyllin A (**17**), hypericorin A (**18**), hypericorin B (**19**), kielcorin (**20**), 3,4,5-trihydroxyxanthone (**21**) and 1,3,7-trihydroxyxanthone (**22**).

MPO levels are considered as a marker of neutrophil activation and oxidative tissue injury (Ibrahim et al., 2018b). Tovophyllin A (**17**), a xanthone isolated from *G. mangostana* L. significantly reduced the hepatic levels of MPO in mice induced with acetaminophen after pre-treated with 50 and 100 mg/kg/day of the xanthone. The effect was linked to the suppression of neutrophil infiltration into the liver (Ibrahim et al., 2018b). Similarly, **2** was reported to inhibit carrageenan-induced inflammation in ICR mice with a reduction of neutrophil infiltration of 82% upon an administration of 25 mg/kg (Mohan et al., 2018). The decrease in the levels of TNF-α and IL-1β in the peritoneal fluids of the mice was simultaneously observed following treatment with **2** (Mohan et al., 2018). Meanwhile, **13** was reported to inhibit the generation of O_2_^•−^ and oxygen consumption induced by formylmethionyl-leucyl-phenylalanine (fMLP) and dihydrocytochalasin B (CB) in rat neutrophils via the concentration-dependent inhibition of phospholipase C (PLC) pathway and NADPH oxidase activity (Hsu et al., 1997). Moreover, other xanthones isolated from the twigs of *Hypericum oblongifolium* Wall. include hypericorin A (**18**), hypericorin B (**19**), kielcorin (**20**), 3,4,5-trihydroxyxanthone (**21**) and 1,3,7-trihydroxyxanthone (**22**) possessed significant inhibition on the production of O_2_^•−^ respiratory burst of neutrophils with IC_50_ values of 816.23, 985.20, 965.21, 907.20 and 975.20 μM, respectively. The chemical structures of **17**-**22** are shown in Figure 3. It was postulated that the potent activity of these xanthones is due to the presence of 1,4-dioxane ring and hydroxyl groups that promote their absorptions into the immune cells (Ali et al., 2011). The capability of these xanthones to decrease neutrophil influx and activity in the areas of inflammation suggested them as suitable drug candidates for psoriasis treatment (Ali et al., 2011).

## Prospects for Future Studies and Applications of Xanthones

The immune response is a complex system with crosstalk between many pathways where the anti-inflammatory activity of xanthones have been revealed to involve a variety of mechanisms. Several targets of xanthones and their signaling pathways such as peroxisome proliferator-activated receptors (PPARs), Nrf2 and prostaglandins have been extensively linked to skin inflammatory diseases. This section focuses on the xanthones that were previously reported to modulate the activity or expression of these molecular targets and their relation to skin inflammatory diseases.

### Peroxisome Proliferator-Activated Receptors as a Target of Xanthones

PPARs and its α, β and γ isoforms have been previously associated with keratinocyte proliferation, differentiation and inflammation (Kim et al., 2006; Sertznig et al., 2008). Downregulation or inactivation of PPAR isoforms disrupts epithelial homeostasis and causes inflammation (Dubrac and Schmuth, 2011). The role of PPARs for skin inflammatory diseases such as psoriasis, skin cancer and acne have been previously reviewed (Sertznig et al., 2008; Sertznig and Reichrath, 2011). Activation of the PPARβ isoform which is also known as PPARδ was reported to selectively initiate terminal differentiation of keratinocytes while simultaneously inhibiting their uncontrolled proliferation (Kim et al., 2006). Additionally, PPARα plays a role in regulating skin inflammation in diseases such as AD and psoriasis by modulating cytokine expression, T cell proliferation, as well as the maturation and migration of Langerhans cells (Dubrac and Schmuth, 2011). Crosstalk with MAPK and NF-κB inflammatory pathways also occurs whereby the activation of these inflammatory pathways results in the inhibition of PPARγ activity (Bumrungpert et al., 2009). Conversely, PPARγ activation antagonizes and inhibits the resulting inflammatory response of NF-κB and AP-1 (Bumrungpert et al., 2010; Mao et al., 2019). Thus, PPAR isoforms were suggested to be a suitable molecular target to treat skin diseases with aberrated cell proliferation such as psoriasis and skin cancers (Kim et al., 2006; Sertznig et al., 2008; Ramot et al., 2015).

Xanthone derivatives such as **2** were found to upregulate PPARγ expression in the preadipocytes in rat retroperitoneal tissue of insulin-resistant mice models following treatment with 5, 10 and 20 mg/kg of the xanthone (Ratwita et al., 2018). Similarly, **3** was shown to act as an agonist of PPARα and PPARδ using a luciferase reporter assay, approximately doubling the luciferase activity in Cos-1 kidney fibroblasts at 2.5 μM (Matsuura et al., 2013). Furthermore, both **2** and **3** when evaluated at 3, 10 and 30 μM restored the mRNA expression of PPARγ that was suppressed by LPS in macrophages (Bumrungpert et al., 2009; Bumrungpert et al., 2010). The restoration of PPARγ expression consequently alleviated inflammation by downregulating the expression of inflammatory cytokines TNF-α, IL-1β and IL-6 (Bumrungpert et al., 2010). Meanwhile, **4** at doses of 10 and 20 mg/kg exhibited cytoprotective effects against oxidative injury in gastric mucosal ischemia/reperfusion mice model via the upregulation of PPARγ expression and concurrent downregulation of NF-κB signaling (Mahmoud-Awny et al., 2015). This anti-inflammatory mechanism exhibited by **4** was further linked to the inhibition of IL-1β, E-selectin and neutrophil infiltration (Mahmoud-Awny et al., 2015). Interestingly, **4** showed no effect on PPAR transactivation whereas its metabolite, **13** successfully inhibited the transactivation of PPARγ, PPARα and PPARβ isoforms in Cos-7 kidney fibroblasts with IC_50_ values of 153.3, 92.8 and 102.4 μM, respectively (Wilkinson et al., 2008). The difference in terms of the ability to transactivate the PPAR isoforms shown by **4** and **13** highlighted the importance of metabolism for the bioactivities of xanthones.

### Nuclear Factor Erythroid 2-Related Factor 2 as a Target of Xanthones

Nrf2 is a transcription factor commonly recognized as the master regulator of cytoprotection and antioxidant defense signaling pathways. This transcription factor is generally activated in response to ROS production and oxidative stress, inducing the downstream expression of antioxidative and phase II detoxification enzymes within the antioxidant response elements (ARE) such as heme oxygenase-1 (HO-1), NAD(P)H quinone oxidoreductase 1 (NQO1), glutathione *S*-transferase, CAT and SOD (Wang et al., 2018a; Helou et al., 2019). The role of oxidative stress and Nrf2 has been extensively implicated with the pathogenesis of skin inflammation and carcinogenesis (Bickers and Athar 2006; Helou et al., 2019; Hennig et al., 2020). Early studies by Braun et al. (2002) identified Nrf2 to be crucial in modulating inflammation in wound healing where persistent and prolonged inflammation associated with IL-1β, IL-6 and TNF-α was observed in Nrf2-knockout mice. Other studies highlighted the anti-inflammatory role of Nrf2 signaling in reducing the levels of IL-1β and IL-6 to alleviate skin inflammation (Saw et al., 2010) besides suppressing the activation of inflammatory NF-κB and NLRP3 pathways (Bulugonda et al., 2017; Helou et al., 2019; Hennig et al., 2020). The Nrf2 signaling pathway was also associated with chemopreventive effects by exerting photoprotection against UVB-induced skin carcinogenesis besides protecting against inflammatory damage on the extracellular matrix (Saw et al., 2010; Saw et al., 2014). Hence, Nrf2 pathway and its central roles in antioxidant defense and anti-inflammation are potential targets in the treatment of various skin inflammatory diseases.

In recent years, the modulatory roles of Nrf2 signaling by xanthones were reported. For example, **4** was reported to activate Nrf2/ARE pathway in response to oxidative stress, inflammation and DNA damage in neurons (Liu et al., 2016), liver (Pan et al., 2016), gastric ulcer models (Mahmoud-Awny et al., 2015) as well as hematopoietic cells (Zhang et al., 2015). Moreover, anti-inflammatory mechanisms of **4** in macrophages also involved the inactivation of NLRP3 inflammasome complex (Bulugonda et al., 2017; Helou et al., 2019). Similarly, **17**, a xanthone isolated from *G. mangostana* L. activated Nrf2 and protected against acetaminophen-induced oxidative stress injury in liver mice models when given 50 and 100 mg/kg/day (Ibrahim et al., 2018b). Other xanthones such as **2** and **3** were also shown to exert antioxidant effects via Nrf2 activation in retinal (Fang et al., 2016) and liver cells (Wang et al., 2018a), respectively.

However, it is also important to understand the possible double-edged effects of Nrf2 activation. In cancer cells, the Nrf2 pathway is upregulated in response to the high ROS production from the increased metabolism of cells (Hambright et al., 2015). Activation of Nrf2 results in cytoprotective effects and prolongs cancer cell survival that in turn antagonizes chemotherapeutic action (Yanaka 2018). Moreover, the constitutive and increased expression of Nrf2 was linked to poor clinical prognosis as it enhances cancer proliferation, angiogenesis, chemoresistance, and radioresistance (Wu et al., 2019). For example, the mRNA and protein expression of Nrf2 in B16-F10 melanoma cells was increased after an exposure to ionizing radiation (Gao et al., 2018). Subsequent knockdown of Nrf2 using small interfering RNAs (siRNAs) coupled with ionizing radiation reduced the migration and invasion of B16-F10 melanoma cells while increasing cellular apoptosis via caspase-3 activity (Gao et al., 2018). Hence, the incorporation of Nrf2 inhibitors in cancer therapy has recently received increasing attention, as reported by Panieri and Saso (2019). In other words, even though Nrf2 pathway is a potential target for skin inflammatory diseases, researchers should be wary on the negative implications in malignancies because an activation of Nrf2 may be beneficial in cytoprotection and chemoprevention whereas the inhibition of Nrf2 may be a valuable strategy in targeting skin malignancies.

### Prostaglandin as a Target of Xanthones

Prostaglandins, also known as eicosanoids, are powerful lipids that modulate immune responses and inflammation (Bull and Dowd, 1993). Several forms of prostaglandins such as prostaglandin E_2_ (PGE_2_) (Bull and Dowd, 1993; Kabashima et al., 2007) and prostaglandin D_2_ (PGD_2_) (Satoh et al., 2006) have been associated with skin inflammation. The enzyme prostaglandin H synthase, which is involved in the synthesis of eicosanoids, generates hydroxyl-endoperoxides that contributes to the co-oxidation of various substrates and the resulting cutaneous inflammation (Bickers and Athar, 2006). Early studies showed the mediatory role of prostaglandins in generating cutaneous inflammation upon UV exposure (Bull and Dowd, 1993). Release of PGE_2_ after UV irradiation subsequently induced histamine secretion from mast cells. Later studies showed the crucial involvement of PGE_2_-EP2/EP4 signaling that resulted in UV-induced acute skin inflammation (Kabashima et al., 2007). This was later supported by Lee et al. (2019) that elucidated the role of PGE_2_-EP2/EP4 signaling in driving inflammation by Th17 cells in IL-23-induced psoriasis mouse models. The underlying involvement of prostaglandins in skin inflammation thus makes them attractive targets for the treatment of inflammatory diseases.

Xanthones were reported to exhibit anti-inflammatory effects by inhibiting the synthesis and release of prostaglandins, as well as downregulating the activity of COX enzymes. For instance, **3** is capable of competitively inhibiting the activities of COX-1 and COX-2 in microsomes of C6 rat glioma cells which consequently prevented the conversion of arachidonic acid to PGE_2_ with an IC_50_ of 17 μM (Nakatani et al., 2002). Subsequent studies reported that **3** suppressed LPS-induced expression of COX-2 at protein and mRNA levels in C6 cells with an IC_50_ of 10 μM while COX-1 expression was not affected (Nakatani et al., 2004). Furthermore, **3** halted the release of PGE_2_ induced by Ca^2+^ ionophores in C6 rat glioma cells in a concentration-dependent manner (IC_50_ = 5 μM) (Nakatani et al., 2002). The antioxidant formulation VIMANG® used in Cuba is an aqueous extract of *Mangifera indica* L. which predominantly consists of **4**. The formulation was shown to be capable of inhibiting PGE_2_ release in stimulated RAW264.7 macrophages with IC_50_ of 64.1 μg/mL (Garrido et al., 2004). Xanthone **4** was isolated and reported to potently inhibit PGE_2_ biosynthesis in RAW264.7 macrophages by 84.3% when treated with 0.024 μM of the compound. In addition, **4** was able to suppress COX-2 expression and production of PGE_2_ in the rat brain when given oral gavages of 30 and 60 mg/kg/day, thereby attenuating inflammation and subsequent oxidative damage (Márquez et al., 2012). Similarly, **13** exerted inhibitory effects on the activities of COX-1, COX-2 as well as lipoxygenases-5 and -12, besides inhibiting the formation of PGD_2_ in neutrophils with IC_50_ values of 16.2, 19.6, 1.8 and 1.2 μM, respectively (Hsu et al., 2004). Thus, **13** was proposed to be a potential therapeutic agent to treat inflammatory diseases due to its multitargeting anti-inflammatory mechanisms.

### Potential Applications of Xanthones for Wound Healing

The application of mangiferin (**4**) in skin wound healing showcased the anti-inflammatory and cytoprotective effects of the xanthone. Recently, 10, 20 and 40 μM of **4** was loaded in hydrogels (Mao et al., 2018) and liposomes (Mao et al., 2019) to enhance random skin flap regeneration which is commonly disrupted by oxidative stress and inflammation-induced apoptosis. Development of delivery systems for **4** solved the issue of hydrophobicity of xanthone which caused aggregation and poor dispersibility of the compound (Mao et al., 2018). This xanthone was found to modulate apoptosis via the Bax/Bcl-2/caspase-3 pathway, exhibiting protective effects by upregulating the Bcl-2/Bax ratio and reducing cleaved caspase-3 expression (Mao et al., 2018). Studies also showed that liposomes loaded with 10, 20 and 40 μM of **4** suppressed the expression and activation of NF-κB while concurrently increasing PPARγ mRNA and protein levels, consequently protecting against inflammation (Mao et al., 2019). Similarly, **4**-hydrogel dose-dependently decreased levels of CD68 and average macrophage density, hence relieving mild local inflammation in a random skin flap animal model (Mao et al., 2018). The observation was supplemented by *in vivo* studies which revealed a decreased necrosis rate and inflammation in a random patterned skin flap rat model, further highlighting the potential wound healing capabilities of **4** (Mao et al., 2019). Besides that, **4** was reported to dose-dependently affect cell proliferation and angiogenesis with an increase of protein expressions of VEGF and bFGF when tested with HUVEC cells (Mao et al., 2018; Mao et al., 2019). However, the observation contradicted the anti-angiogenic effect of **4** on B16-F10 melanoma cells as discussed earlier. The discrepancy may be linked to cell-specific effects which suggests **4** to be a highly versatile compound for numerous potential applications in skin disorders.

### Perspectives for Xanthone in Skin Inflammatory Diseases Research

The ideal therapeutic approach, whether single-targeting or multitargeting, is still being debated within the scientific community. Numerous publications to date discuss the shifting paradigm in drug development (Talevi, 2015; Ramsay et al., 2018). The “one drug, one target” strategy is traditionally associated with high selectivity and hence lower risks of off-target effects (Ramsay et al., 2018). This concept has been the basis of drug development for decades and resulted in immeasurable success for the pharmaceutical industry. Nonetheless, the efficacy of single-targeting drugs may be limited against complex and multifactorial diseases due to compensatory mechanisms or overlapping biological functions in immune pathophysiology (Makhoba et al., 2020). Thus, the concept of polypharmacology which aims to develop single drugs that recognize multiple molecular targets emerged rapidly as a popular strategy in drug design and development (Bolognesi, 2013; Makhoba et al., 2020). Moreover, 21% of new drugs approved by the Food and Drug Administration (FDA) between 2015 to 2017 were multitarget drugs (Ramsay et al., 2018).

In relation to skin inflammatory diseases, multitargeting mechanisms of action may be advantageous for diseases such as acne and skin malignancies due to the development of drug resistance. For instance, single-targeting mechanisms are ineffective in cancer therapeutics to counter cancer proliferation and metastasis (Delgado-Hernández et al., 2019). However, it must be acknowledged that with an increase in the number of targets, the risk of off-target effects simultaneously increases. The advantages and disadvantages of single targeting versus multitargeting drugs in the context of skin inflammatory diseases have yet to fully addressed. However, the complexity and overlapping immune responses relating to skin inflammatory diseases implied that multitargeting compounds such as xanthones may have good potential in the treatment of these diseases.

Nevertheless, despite the advancing research of xanthones for skin inflammatory diseases, the molecular mechanisms of action are still not completely elucidated. Furthermore, numerous studies do not move past experiments using *in vivo* mice models. Minimal data on the pharmacokinetic and pharmacodynamic properties of xanthones including their bioavailability and safety profile thus limit the progression of xanthones through the drug development process (Ng and Chua, 2019). In addition, the issue of bioavailability has also driven the researchers to develop drug carriers or formulations to improve xanthone delivery to the target site (Asasutjarit et al., 2013; Pan-In et al., 2015; Mao et al., 2018; Pleguezuelos-Villa et al., 2019). These studies are nonetheless still limited and have great rooms to be researched on, thus further studies are recommended to fill these gaps.

## Conclusion

Xanthones possess a wide range of biological properties such as antioxidant, anti-inflammatory and chemotherapeutic effects that are effective in treating various skin diseases such as acne, atopic dermatitis, psoriasis, and skin cancer. However, the modulatory effects of xanthones on other mediators of inflammation such as PPARs, Nrf2 and prostaglandins have yet to be thoroughly explored in skin inflammatory diseases despite publications highlighting these mediators to be potential molecular targets. As privileged structures and versatile scaffolds, xanthones such as α-mangostin (**2**), γ-mangostin (**3**), mangiferin (**4**) and gambogic acid (**8**) are potential lead compounds to be further developed into pharmaceutical agents for skin inflammatory diseases. Prospective applications of xanthones are vast with synthetic opportunities remaining uncharted. Further studies on the structure-activity relationships, molecular mechanisms, and applications of xanthones for the treatment of skin inflammatory diseases are recommended.

## Author Contributions

NG, ST, YL, and SM contributed to the idea and drafted the manuscript. All authors contributed to the article and approved the submitted version.

## Funding

Fundamental Research Grant Scheme (FRGS) (FRGS/1/2019/STG01/TAYLOR/02/1) awarded to SM from the Ministry of Education (MOE) is acknowledged.

## Conflict of Interest

The authors declare that the research was conducted in the absence of any commercial or financial relationships that could be construed as a potential conflict of interest.

## References

[B1] Aizat W. M. Jamil I. N. Ahmad-Hashim F. H. Noor N. M. (2019). Recent updates on metabolite composition and medicinal benefits of mangosteen plant. PeerJ 7, e6324 10.7717/peerj.6324 30755827 PMC6368837

[B2] Ali M. Arfan M. Ahmad M. Singh K. Anis I. Ahmad H. (2011). Anti-inflammatory xanthones from the twigs of *Hypericum oblongifolium* wall. Planta Med. 77 (18), 2013–2018. 10.1055/s-0031-1280114 21870324

[B3] Alperth F. Mitić B. Mayer S. Maleš Ž. Kunert O. Hruševar D. (2018). Metabolic profiling of rhizomes of native populations of the strictly endemic *Croatian* species *Iris adriatica* . Plant Biosys. 153 (2), 317–324. 10.1080/11263504.2018.1478906

[B4] Asasutjarit R. Larpmahawong P. Fuongfuchat A. Sareedenchai V. Veeranondha S. (2013). Physicochemical properties and anti-*Propionibacterium acnes* activity of film-forming solutions containing alpha-mangostin-rich extract. AAPS PharmSciTech 15 (2), 306–316. 10.1208/s12249-013-0057-8 24327275 PMC3969485

[B5] Asasutjarit R. Meesomboon T. Adulheem P. Kittiwisut S. Sookdee P. Samosornsuk W. (2019). Physicochemical properties of alpha-mangostin loaded nanomeulsions prepared by ultrasonication technique. Heliyon 5 (9), e02465 10.1016/j.heliyon.2019.e02465 31538120 PMC6745438

[B6] Auranwiwat C. Trisuwan K. Saiai A. Pyne S. G. Ritthiwigrom T. (2014). Antibacterial tetraoxygenated xanthones from the immature fruits of *Garcinia cowa* . Fitoterapia 98, 179–183. 10.1016/j.fitote.2014.08.003 25110196

[B7] Auriemma M. Vianale G. Amerio P. Reale M. (2013). Cytokines and T cells in atopic dermatitis. Eur. Cytokine Netw. 24 (1), 37–44. 10.1684/ecn.2013.0333 23608610

[B8] Aye A. Song Y. J. Jeon Y. D. Jin J. S. (2020). Xanthone suppresses allergic contact dermatitis *in vitro* and *in vivo* . Int. Immunopharm. 78 (106061). 10.1016/j.intimp.2019.106061 31821937

[B9] Beninati S. Oliverio S. Cordella M. Rossi S. Senatore C. Liguori I. (2014). Inhibition of cell proliferation, migration and invasion of B16-F10 melanoma cells by α-mangostin. Biochem. Biophys. Res. Commun. 450 (4), 1512–1517. 10.1016/j.bbrc.2014.07.031 25019992

[B10] Bhaskar G. Arshia S. Priyadarshini S. (2009). Formulation and evaluation of topical polyherbal antiacne gels containing *Garcinia mangostana* and *Aloe vera* . Phcog. Mag. 5 (19), 93–99.

[B11] Biasi D. Carletto A. Caramaschi P. Bellavite P. Maleknia T. Scambi C. (1998). Neutrophil functions and IL-8 in psoriatic arthritis and in cutaneous psoriasis. Inflammation 22 (5), 533–543. 10.1023/a:1022354212121 9793799

[B12] Bickers D. R., Athar M. (2006). Oxidative stress in the pathogenesis of skin disease. J. Invest. Dermatol. 126 (12), 2565–2575. 10.1038/sj.jid.5700340 17108903

[B13] Bolognesi M. L. (2013). Polypharmacology in a single drug: multitarget drugs. Curr. Med. Chem. 20 (13), 1639–1645. 10.2174/0929867311320130004 23410164

[B14] Braun S. Hanselmann C. Gassmann M. G. auf dem Keller U. Born-Berclaz C. Chan K. (2002). Nrf2 transcription factor, a novel target of keratinocyte growth factor action which regulates gene expression and inflammation in the healing skin wound. Mol. Cell. Biol. 22 (15), 5492–5505. 10.1128/mcb.22.15.5492-5505.2002 12101242 PMC133949

[B15] Buddenkotte J. Maurer M. Steinhoff M. (2010). Histamine and antihistamines in atopic dermatitis. Adv. Exp. Med. Biol. 709, 73–80. 10.1007/978-1-4419-8056-4_8 21618889

[B16] Bull H. A., Dowd P. M. (1993). Prostaglandins, interleukins, and cutaneous inflammation. Immunomethods 2 (3), 219–226. 10.1006/immu.1993.1025

[B17] Bulugonda R. K. Kumar K. A. Gangappa D. Beeda H. Philip G. H. Rao D. D. (2017). Mangiferin from *Pueraria tuberosa* reduces inflammation *via* inactivation of NLRP3 inflammasome. Sci. Rep. 7, 42683 10.1038/srep42683 28218280 PMC5316935

[B18] Bumrungpert A. Kalpravidh R. W. Chitchumroonchokchai C. Chuang C.-C. West T. Kennedy A. (2009). Xanthones from mangosteen prevent lipopolysaccharide-mediated inflammation and insulin resistance in primary cultures of human adipocytes. J. Nutr. 139 (6), 1185–1191. 10.3945/jn.109.106617 19403722

[B19] BumrungpertA.KalpravidhR. W.ChuangC.-C.OvermanA.MartinezK.KennedyA. (2010). Xanthones from mangosteen inhibit inflammation in human macrophages and in human adipocytes exposed to macrophage-conditioned media. J. Nutr. 140 (4), 842–847. 10.3945/jn.109.120022 20181789

[B20] Campbell K. J., Tait S. W. G. (2018). Targeting BCL-2 regulated apoptosis in cancer. Open Biol. 8 (5), 180002 10.1098/rsob.180002 29769323 PMC5990650

[B21] Chairungsrilerd N. Furukawa K.-I. Ohta T. Nozoe S. Ohizumi Y. (1996b). Histaminergic and serotonergic receptor blocking substances from the medicinal PlantGarcinia mangostana. Planta Med. 62 (5), 471–472. 10.1055/s-2006-957943 8923814

[B22] Chairungsrilerd N. Furukawa K.-I. Ohta T. Nozoe S. Ohizumi Y. (1996a). Pharmacological properties of α-mangostin, a novel histamine H1 receptor antagonist. Eur. J. Pharmacol. 314 (3), 351–356. 10.1016/s0014-2999(96)00562-6 8957258

[B23] Cheng H. Zhang X. Su J. J. Li Q. L. (2014). Study of gambogenic acid-induced apoptosis of melanoma B16 cells through PI3K/Akt/mTOR signaling pathways. Zhongguo Zhongyao Zazhi 39 (9), 1666–1669 [in Chinese]. 25095381

[B24] Chiu Y.-S. Wu J.-L. Yeh C.-T. Yadav V. K. Huang H.-S. Wang L.-S. (2020). γ-Mangostin isolated from *Garcinia mangostana* L. suppresses inflammation and alleviates symptoms of osteoarthritis via modulating miR-124-3p/IL-6/NF-κB signaling. Aging 12 (8), 6630–6643. 10.18632/aging.103003 32302289 PMC7202528

[B25] Chomnawang M. T. Surassmo S. Nukoolkarn V. S. Gritsanapan W. (2005). Antimicrobial effects of Thai medicinal plants against acne-inducing bacteria. J. Ethnopharmacol. 101 (1–3), 330–333. 10.1016/j.jep.2005.04.038 16009519

[B26] Chomnawang M. T. Surassmo S. Nukoolkarn V. S. Gritsanapan W. (2007). Effect of *Garcinia mangostana* on inflammation caused by *Propionibacterium acnes* . Fitoterapia 78 (6), 401–408. 10.1016/j.fitote.2007.02.019 17644272

[B27] Cidade H. Rocha V. Palmeira A. Marques C. Tiritan M. E. Ferreira H. (2017). *In silico* and *in vitro* antioxidant and cytotoxicity evaluation of oxygenated xanthone derivatives. Arab. J. Chem. 13 (1), 17–26. 10.1016/j.arabjc.2017.01.006

[B28] Delgado-Hernández R. Hernández-Balmaseda I. Rodeiro-Guerra I. Gonzalez J. C. R. De Wever O. Logie E. (2019). Anti-angiogenic effects of mangiferin and mechanism of action in metastatic melanoma. Melanoma Res. 30 (1), 39–51. 10.1097/CMR.0000000000000647 31651714

[B29] Dubrac S., Schmuth M. (2011). PPAR-alpha in cutaneous inflammation. Derm. Endocrinol. 3 (1), 23–26. 10.4161/derm.3.1.14615 PMC3051849 21519405

[B30] Fang Y. Su T. Qiu X. Mao P. Xu Y. Hu Z. (2016). Protective effect of alpha-mangostin against oxidative induced-retinal cell death. Sci. Rep. 6, 21018 10.1038/srep21018 26888416 PMC4757868

[B31] Feng Z. Lu X. Gan L. Zhang Q. Lin L. (2020). Xanthones, a promising anti-inflammatory scaffold: structure, activity, and drug likeness analysis. Molecules 25 (3), 598 10.3390/molecules25030598 PMC7037265 32019180

[B32] Gao Y. Zhao Z. Meng X. Chen H. Fu G. (2018). Migration and invasion in B16-F10 mouse melanoma cells are regulated by Nrf2 inhibition during treatment with ionizing radiation. Oncol. Lett. 16 (2), 1959–1966. 10.3892/ol.2018.8799 30008889 PMC6036499

[B33] Garrido G. González D. Lemus Y. García D. Lodeiro L. Quintero G. (2004). *In vivo* and *in vitro* anti-inflammatory activity of *Mangifera indica* L. extract (VIMANGS). Pharmacol. Res. 50 (2), 143–149. 10.1016/j.phrs.2003.12.003 15177302

[B34] Guérard S. Allaeys I. Martin G. Pouliot R. Poubelle P. E. (2013). Psoriatic keratinocytes prime neutrophils for an overproduction of superoxide anions. Arch. Dermatol. Res. 305 (10), 879–889. 10.1007/s00403-013-1404-z 23974213

[B35] Guo H.-W. Yun C.-X. Hou G.-H. Du J. Huang X. Lu Y. (2014). Mangiferin attenuates Th1/Th2 cytokine imbalance in an ovalbumin-induced asthmatic mouse model. PLoS One 9 (6), e100394 10.1371/journal.pone.0086881 24955743 PMC4067356

[B36] Guo H. Liu H. Jian Z. Cui H. Fang J. Zuo Z. (2019). Nickel induces inflammatory activation via NF-κB, MAPKs, IRF3 and NLRP3 inflammasome signaling pathways in macrophages. Aging 11 (23), 11659–11672. 10.18632/aging.102570 31822637 PMC6932914

[B37] Gutierrez-Orozco F. Chitchumroonchokchai C. Lesinski G. B. Suksamrarn S. Failla M. L. (2013). α-Mangostin: anti-inflammatory activity and metabolism by human cells. J. Agric. Food Chem. 61 (16), 3891–3900. 10.1021/jf4004434 23578285 PMC3793015

[B38] Hambright H. G. Meng P. Kumar A. P. Ghosh R. (2015). Inhibition of PI3K/AKT/mTOR axis disrupts oxidative stress-mediated survival of melanoma cells. Oncotarget 6 (9), 7195–7208. 10.18632/oncotarget.3131 25749517 PMC4466678

[B39] Heinze H., Mohr H. (1990). “Phorbol myristate acetate and calcium ionophore A23187 induce two waves of lymphokine mRNA transcription in stimualted human peripheral blood lymphocytes,”in Cytokines in hemopoiesis, oncology, and AIDS. Editors Freund M. Link H. Welte K. (Berlin, Heidelberg: Springer), 359–363.

[B40] Helou D. G. Martin S. F. Pallardy M. Chollet-Martin S. Kerdine-Römer S. (2019). Nrf2 involvement in chemical-induced skin innate immunity. Front. Immunol. 10, 1004 10.3389/fimmu.2019.01004 31134077 PMC6514534

[B41] Heng A. H. S., Chew F. T. (2020). Systematic review of the epidemiology of acne vulgaris. Sci. Rep. 10 (1), 5754 10.1038/s41598-020-62715-3 32238884 PMC7113252

[B42] Hennig P. Fenini G. Di Filippo M. Beer H. D. (2020). Electrophiles against (skin) diseases: more than Nrf2. Biomolecules 10 (2), 271 10.3390/biom10020271 PMC7072181 32053878

[B43] Herrera-Aco D. R. Medina-Campos O. N. Pedraza-Chaverri J. Sciutto-Conde E. Rosas-Salgado G. Fragoso-González G. (2019). Alpha-mangostin: anti-inflammatory and antioxidant effects on established collagen-induced arthritis in DBA/1J mice. Food Chem. Toxicol. 124, 300–315. 10.1016/j.fct.2018.12.018 30557668

[B44] Higuchi H. Tanaka A. Nishikawa S. Oida K. Matsuda A. Jung K. (2013). Suppressive effect of mangosteen rind extract on the spontaneous development of atopic dermatitis in NC/Tnd mice. J. Dermatol. 40 (10), 786–796. 10.1111/1346-8138.12250 24033377

[B45] Hirakawa K. Yoshida M. Nagatsu A. Mizukami H. Rana V. Rawat M. S. M. (2005). Chemopreventive action of xanthone derivatives on photosensitized DNA damage¶. Photochem. Photobiol. 81 (2), 314–319. 10.1562/2004-07-29-ra-252.1 15646999

[B46] Hiraku Y. Ito K. Hirakawa K. Kawanishi S. (2007). Photosensitized DNA damage and its protection via a novel mechanism. Photochem. Photobiol. 83 (1), 205–212. 10.1562/2006-03-09-ir-840 16965181

[B47] Hsu M.-F. Lin C.-N. Lu M.-C. Wang J.-P. (2004). Inhibition of the arachidonic acid cascade by norathyriol via blockade of cyclooxygenase and lipoxygenase activity in neutrophils. N. Schmied. Arch. Pharmacol. 369 (5), 507–515. 10.1007/s00210-004-0922-9 15083266

[B48] Hsu M.-F. Raung S.-L. Tsao L.-T. Lin C.-N. Wang J.-P. (1997). Examination of the inhibitory effect of norathyriol in formylmethionyl-leucyl-phenylalanine-induced respiratory burst in rat neutrophils. Free Radic. Biol. Med. 23 (7), 1035–1045. 10.1016/s0891-5849(97)00132-9 9358247

[B49] Ibrahim M. Y. Hashim N. M. Mariod A. A. Mohan S. Abdulla M. A. Abdelwahab S. I. (2016). α-Mangostin from *Garcinia mangostana* Linn: an updated review of its pharmacological properties. Arab. J. Chem. 9 (3), 317–329. 10.1016/j.arabjc.2014.02.011

[B50] Ibrahim S. R. M. Abdallah H. M. El-Halawany A. M. Nafady A. M. Mohamed G. A. (2018a). Mangostanaxanthone VIII, a new xanthone from *Garcinia mangostana* and its cytotoxic activity. Nat. Prod. Res. 33 (2), 258–265. 10.1080/14786419.2018.1446012 29513040

[B51] Ibrahim S. R. M. El-Agamy D. S. Abdallah H. M. Ahmed N. Elkablawy M. A. Mohamed G. A. (2018b). Protective activity of tovophyllin A, a xanthone isolated from *Garcinia mangostana* pericarps, against acetaminophen-induced liver damage: role of Nrf2 activation. Food Funct. 9 (6), 3291–3300. 10.1039/c8fo00378e 29790527

[B52] Im A. R. Kim Y. M. Chin Y. W. Chae S. (2017). Protective effects of compounds from *Garcinia mangostana* L. (Mangosteen) against UVB damage in HaCaT cells and hairless mice. Int. J. Mol. Med. 40 (6), 1941–1949. 10.3892/ijmm.2017.3188 29039482

[B53] Jang H.-Y. Kwon O.-K. Oh S.-R. Lee H.-K. Ahn K.-S. Chin Y.-W. (2012). Mangosteen xanthones mitigate ovalbumin-induced airway inflammation in a mouse model of asthma. Food Chem. Toxicol. 50 (11), 4042–4050. 10.1016/j.fct.2012.08.037 22943973

[B54] Kabashima K. Nagamachi M. Honda T. Nishigori C. Miyachi Y. Tokura Y. (2007). Prostaglandin E2 is required for ultraviolet B-induced skin inflammation via EP2 and EP4 receptors. Lab. Invest. 87 (1), 49–55. 10.1038/labinvest.3700491 17075575

[B55] Kalz F. (1960). The effect of prolonged therapy of atopic dermatitis with triamcinolone, dexamethasone and 6-methyl prednisolone. Dermatology 120, 65–74. 10.1159/000255301 14404209

[B56] Kapadia G. J. S. Rao G. Takayasu J. Takasaki M. Iida A. Suzuki N. (2013). Evaluation of skin cancer chemoprevention potential of sunscreen agents using the Epstein-Barr virus early antigen activationin vitroassay. Int. J. Cosmet. Sci. 35 (2), 143–148. 10.1111/ics.12015 23075132

[B57] Khazaei Z. Ghorat F. Jarrahi A. M. Adineh H. A. Sohrabivafa M. Goodarzi E. (2019). Global incidence and mortality of skin cancer by histological subtype and its relationship with the human development index (HDI); an ecology study in 2018. World Cancer Res. J. 6, e1265 10.32113/wcrj_20194_1265

[B58] Khmaladze I. Nandakumar K. S. Holmdahl R. (2015). Reactive oxygen species in psoriasis and psoriasis arthritis: relevance to human disease. Int. Arch. Allergy Immunol. 166 (2), 135–149. 10.1159/000375401 25824670

[B59] Kim D. J. Bility M. T. Billin A. N. Willson T. M. Gonzalez F. J. Peters J. M. (2006). PPARβ/δ selectively induces differentiation and inhibits cell proliferation. Cell Death Differ. 13 (1), 53–60. 10.1038/sj.cdd.4401713 16021179

[B60] Koh J.-J. Qiu S. Zou H. Lakshminarayanan R. Li J. Zhou X. (2013). Rapid bactericidal action of alpha-mangostin against MRSA as an outcome of membrane targeting. Biochim. Biophys. Acta Biomembr. 1828 (2), 834–844. 10.1016/j.bbamem.2012.09.004 22982495

[B61] Kuete V. Wabo H. K. Eyong K. O. Feussi M. T. Wiench B. Krusche B. (2011). Anticancer activities of six selected natural compounds of some Cameroonian medicinal plants. PLoS One 6 (8), e21762 10.1371/journal.pone.0021762 21886765 PMC3158745

[B62] Lee D.-J. Du F. Chen S.-W. Nakasaki M. Rana I. Shih V. F. S. (2015). Regulation and function of the caspase-1 in an inflammatory microenvironment. J. Invest. Dermatol. 135 (8), 2012–2020. 10.1038/jid.2015.119 25815426 PMC4504759

[B63] Lee J. Aoki T. Thumkeo D. Siriwach R. Yao C. Narumiya S. (2019). T cell-intrinsic prostaglandin E2-EP2/EP4 signaling is critical in pathogenic Th17 cell-driven inflammation. J. Allergy Clin. Immunol. 143 (2), 631–643. 10.1016/j.jaci.2018.05.036 29935220 PMC6354914

[B64] Lee L.-T. Tsai Y.-F. Hu N.-Y. Wang C.-W. Huang K.-K. Hsiao J.-K. (2013). Anti-arthritis effect of mangostins from *G. Mangostana* . Biomed. Prevent. Nutr. 3 (3), 227–232. 10.1016/j.bionut.2012.10.002

[B65] Li J. Malakhova M. Mottamal M. Reddy K. Kurinov I. Carper A. (2012). Norathyriol suppresses skin cancers induced by solar ultraviolet radiation by targeting ERK kinases. Cancer Res. 72 (1), 260–270. 10.1158/0008-5472.can-11-2596 22084399 PMC3251698

[B66] Liang L. Zhang Z. (2016). Gambogic acid inhibits malignant melanoma cell proliferation through mitochondrial p66shc/ROS-p53/Bax-mediated apoptosis. Cell. Physiol. Biochem. 38 (4), 1618–1630. 10.1159/000443102 27119348

[B67] Liang L. Zhang Z. Qin X. Gao Y. Zhao P. Liu J. (2018). Gambogic acid inhibits melanoma through regulation of miR-199a-3p/ZEB1 signalling. Basic Clin. Pharmacol. Toxicol. 123 (6), 692–703. 10.1111/bcpt.13090 29959879

[B68] Liu Y.-W. Cheng Y.-Q. Liu X.-L. Hao Y.-C. Li Y. Zhu X. (2016). Mangiferin upregulates glyoxalase 1 through activation of Nrf2/ARE signaling in central neurons cultured with high glucose. Mol. Neurobiol. 54 (6), 4060–4070. 10.1007/s12035-016-9978-z 27318675

[B69] Lopez Carrera I. Al Hammadi A. Huang Y. H. Llamado L. J. Mahgoub E. Tallman A. M. (2019). Epidemiology, diagnosis, and treatment of atopic dermatitis in the developing countries of Asia, Africa, Latin America, and the Middle East: a review. Dermatol. Ther. 9 (4), 685–705. 10.1007/s13555-019-00332-3 PMC6828917 31650504

[B70] Magcwebeba T. U. Riedel S. Swanevelder S. Swart P. De Beer D. Joubert E. (2016a). The potential role of polyphenols in the modulation of skin cell viability by *Aspalathus linearis* and *Cyclopia* spp. herbal tea extracts *in vitro* . J. Pharm. Pharmacol. 68 (11), 1440–1453. 10.1111/jphp.12629 27671741

[B71] Magcwebeba T. U. Swart P. Swanevelder S. Joubert E. Gelderblom W. C. A. (2016b). Anti-inflammatory effects of *Aspalathus linearis* and *Cyclopia* spp. extracts in a UVB/keratinocyte (HaCaT) model utilising interleukin-1α accumulation as biomarker. Molecules 21 (10), 1323 10.3390/molecules21101323 PMC6274390 27706097

[B72] Mah S. Teh S. Lian Ee G. (2019). Comparative studies of selected *Calophyllum* plants for their anti-inflammatory properties. Phcog. Mag. 15 (60), 135–139. 10.4103/pm.pm_212_18

[B73] Mahmoud-Awny M. Attia A. S. Abd-Ellah M. F. El-Abhar H. S. (2015). Mangiferin mitigates gastric ulcer in ischemia/reperfused rats: involvement of PPAR-γ, NF-κB and Nrf2/HO-1 signaling pathways. PLoS One 10 (7), e0132497 10.1371/journal.pone.0132497 26196679 PMC4509761

[B74] Makhoba X. H. Viegas C. Jr. Mosa R. A. Viegas F. P. Pooe O. J. (2020). Potential impact of the multi-target dug approach in the treatment of some complex diseases. Drug Des. Dev. Ther. 14, 3235–3249. 10.2147/dddt.s257494 PMC7440888 32884235

[B75] Mao X. Cheng R. Zhang H. Bae J. Cheng L. Zhang L. (2018). Self-healing and injectable hydrogel for matching skin flap regeneration. Adv. Sci. 6 (13), 1801555 10.1002/advs.201801555 PMC6364594 30775235

[B76] Mao X. Liu L. Cheng L. Cheng R. Zhang L. Deng L. (2019). Adhesive nanoparticles with inflammation regulation for promoting skin flap regeneration. J. Contr. Release 297, 91–101. 10.1016/j.jconrel.2019.01.031 30690104

[B77] Mariette X. Matucci-Cerinic M. Pavelka K. Taylor P. van Vollenhoven R. Heatley R. (2011). Malignancies associated with tumour necrosis factor inhibitors in registries and prospective observational studies: a systematic review and meta-analysis. Ann. Rheum. Dis. 70 (11), 1895–1904. 10.1136/ard.2010.149419 21885875

[B78] Márquez L. García-Bueno B. Madrigal J. L. M. Leza J. C. (2012). Mangiferin decreases inflammation and oxidative damage in rat brain after stress. Eur. J. Nutr. 51 (6), 729–739. 10.1007/s00394-011-0252-x 21986672

[B79] Matsuura N. Gamo K. Miyachi H. Iinuma M. Kawada T. Takahashi N. (2013). γ-mangostin from *Garcinia mangostana* pericarps as a dual agonist that activates both PPARα and PPARδ *Bioscience* . Biotechnol. Biochem. 77 (12), 2430–2435. 10.1271/bbb.130541 24317060

[B80] Mercer L. K. Askling J. Raaschou P. Dixon W. G. Dreyer L. Hetland M. L. (2017). Risk of invasive melanoma in patients with rheumatoid arthritis treated with biologics: results from a collaborative project of 11 European biologic registers. Ann. Rheum. Dis. 76 (2), 386–391. 10.1136/annrheumdis-2016-209285 27307502 PMC5284347

[B81] Mohan S. Syam S. Abdelwahab S. I. Thangavel N. (2018). An anti-inflammatory molecular mechanism of action of α-mangostin, the major xanthone from the pericarp of *Garcinia mangostana*: an *in silico, in vitro* and *in vivo* approach. Food Funct. 9 (7), 3860–3871. 10.1039/c8fo00439k 29953154

[B82] Nakatani K. Nakahata N. Arakawa T. Yasuda H. Ohizumi Y. (2002). Inhibition of cyclooxygenase and prostaglandin E2 synthesis by γ-mangostin, a xanthone derivative in mangosteen, in C6 rat glioma cells. Biochem. Pharmacol. 63 (1), 73–79. 10.1016/s0006-2952(01)00810-3 11754876

[B83] Nakatani K. Yamakuni T. Kondo N. Arakawa T. Oosawa K. Shimura S. (2004). γ-mangostin inhibits inhibitor-κB kinase activity and decreases lipopolysaccharide-induced cyclooxygenase-2 gene expression in C6 rat glioma cells. Mol. Pharmacol. 66 (3), 667–674. 10.1124/mol.104.002626 15322259

[B84] Ng I. M. J. Chua C. L. L. (2019). The potential of xanthones as a therapeutic option in macrophage-associated inflammatory diseases. Phcog. Rev. 13 (25), 28–33. 10.4103/phrev.phrev_25_18

[B85] Ng I. M. J. Mah S. H. Chua C. L. L. (2020). Immuno-modulatory effects of macluraxanthone on macrophage phenotype and function. Nat. Prod. Res. 1–6. 10.1080/14786419.2020.1775223 32508145

[B86] Ohsawa Y. Hirasawa N. (2014). The role of histamine H1 and H4 receptors in atopic dermatitis: from basic research to clinical study. Allergol. Int. 63 (4), 533–542. 10.2332/allergolint.13-ra-0675 25249063

[B87] Okayama Y. (2005). Oxidative stress in allergic and inflammatory skin diseases. Curr. Drug Targets - Inflamm. Allergy 4 (4), 517–519. 10.2174/1568010054526386 16127829

[B88] Ong Y. S. Murugaiyah V. Goh B. H. Khaw K. Y. (2020). “Bioactive xanthones from Garcinia mangostana,” in Plant-derived Bioactives. Editors Swamy M. (Singapore, Singapore: Springer), 281–300.

[B89] Owczarek K. Jaworski M. (2016). Quality of life and severity of skin changes in the dynamics of psoriasis. Adv. Dermatol. Allergol. 33 (2), 102–108. 10.5114/pdia.2015.54873 PMC4884773 27279818

[B90] Pan C. W. Pan Z. Z. Hu J. J. Chen W. L. Zhou G. Y. Lin W. (2016). Mangiferin alleviates lipopolysaccharide and D-galactosamine-induced acute liver injury by activating the Nrf2 pathway and inhibiting NLRP3 inflammasome activation. Eur. J. Pharmacol. 770, 85–91. 10.1016/j.ejphar.2015.12.006 26668000

[B91] Pan-In P. Wongsomboon A. Kokpol C. Chaichanawongsaroj N. Wanichwecharungruang S. (2015). Depositing alpha-mangostin nanoparticles to sebaceous gland area for acne treatment. J. Pharmacol. Sci. 129 (4), 226–232. 10.1016/j.jphs.2015.11.005 26701606

[B92] Panieri E. Saso L. (2019). Potential applications of Nrf2 inhibitors in cancer therapy. Oxidat. Med. Cell. Longev. 2019, 8592348 10.1155/2019/8592348 PMC6487091 31097977

[B93] Pedraza-Chaverri J. Reyes-Fermín L. M. Nolasco-Amaya E. G. Orozco-Ibarra M. Medina-Campos O. N. Gonzalez-Cuahutencos O. (2009). ROS scavenging capacity and neuroprotective effect of α-mangostin against 3-nitropropionic acid in cerebellar granule neurons. Exp. Toxicol. Pathol. 61 (5), 491–501. 10.1016/j.etp.2008.11.002 19108999

[B94] Pérez-Rojas J. M. Cruz C. García-López P. Sánchez-González D. J. Martínez-Martínez C. M. Ceballos G. (2009). Renoprotection by α-mangostin is related to the attenuation in renal oxidative/nitrosative stress induced by cisplatin nephrotoxicity. Free Radic. Res. 43 (11), 1122–1132. 10.1080/10715760903214447 19863372

[B95] Petrova A. Davids L. M. Rautenbach F. Marnewick J. L. (2011). Photoprotection by honeybush extracts, hesperidin and mangiferin against UVB-induced skin damage in SKH-1 mice. J. Photochem. Photobiol. B Biol. 103 (2), 126–139. 10.1016/j.jphotobiol.2011.02.020 21435898

[B96] Pittayapruek P. Meephansan J. Prapapan O. Komine M. Ohtsuki M. (2016). Role of matrix metalloproteinases in photoaging and photocarcinogenesis. Int. J. Mol. Sci. 17 (6), 868 10.3390/ijms17060868 PMC4926402 27271600

[B97] Pleguezuelos-Villa M. Diez-Sales O. Manca M. L. Manconi M. Sauri A. R. Escribano-Ferrer E. (2019). Mangiferin glycethosomes as a new potential adjuvant for the treatment of psoriasis. Int. J. Pharm. 573, 118844 10.1016/j.ijpharm.2019.118844 31751638

[B98] Pothitirat W. Chomnawang M. T. Supabphol R. Gritsanapan W. (2010). Free radical scavenging and anti-acne activities of mangosteen fruit rind extracts prepared by different extraction methods. Pharmaceut. Biol. 48 (2), 182–186. 10.3109/13880200903062671 20645837

[B99] Purnaningsih D. Djawad K. Wahab S. Massi N. Alam G. Bahar B. (2018). Protective effect of mangosteen skin extract on albino mice skin with dimethyl-benz(α)anthracene (DMBA) induction: analysis on p53 protein level. Int. J. Med. Rev. Case Rep. 3, 213–215.

[B100] Ramot Y. Mastrofrancesco A. Camera E. Desreumaux P. Paus R. Picardo M. (2015). The role of PPARγ-mediated signalling in skin biology and pathology: new targets and opportunities for clinical dermatology. Exp. Dermatol. 24 (4), 245–251. 10.1111/exd.12647 25644500

[B101] Ramsay R. R. Popovic-Nikolic M. R. Nikolic K. Uliassi E. Bolognesi M. L. (2018). A perspective on multi-target drug discovery and design for complex diseases. Clin. Transl. Med. 7 (1), 3 10.1186/s40169-017-0181-2 29340951 PMC5770353

[B102] Ratwita W. Sukandar E. Y. Kurniati N. F. Adnyana I. K. (2018). Alpha mangostin and xanthone from mangosteen (*Garcinia mangostana* L.) role on insulin tolerance and PPAR-γ in preclinical model diabetes mellitus. J. Pharm. Nutr. Sci. 8, 83–90. 10.6000/1927-5951.2018.08.03.1

[B103] Rayburn E. R. Ezell S. J. Zhang R. (2009). Anti-inflammatory agents for cancer therapy. Mol. Cell. Pharmacol. 1 (1), 29–43. 10.4255/mcpharmacol.09.05 20333321 PMC2843097

[B104] Ruan J. Zheng C. Liu Y. Qu L. Yu H. Han L. (2017). Chemical and biological research on herbal medicines rich in xanthones. Molecules 22 (10), 1698 10.3390/molecules22101698 PMC6151445 29019929

[B105] Sankowski A. J. Łebkowska U. M. Ćwikła J. Walecka I. Walecki J. (2013). Psoriatic arthritis. Pol. J. Radiol. 78 (1), 7–17. 10.12659/PJR.883763 PMC3596149 23493653

[B106] Satoh T. Moroi R. Aritake K. Urade Y. Kanai Y. Sumi K. (2006). Prostaglandin D2 plays an essential role in chronic allergic inflammation of the skin via CRTH2 receptor. J. Immunol. 177 (4), 2621–2629. 10.4049/jimmunol.177.4.2621 16888024

[B107] Saw C. L. Huang M. T. Liu Y. Khor T. O. Conney A. H. Kong A. N. (2010). Impact of Nrf2 on UVB-induced skin inflammation/photoprotection and photoprotective effect of sulforaphane. Mol. Carcinog. 50 (6), 479–486. 10.1002/mc.20725 21557329

[B108] Saw C. L. Yang A. Y. Huang M. T. Liu Y. Lee J. H. Khor T. O. (2014). Nrf2 null enhances UVB-induced skin inflammation and extracellular matrix damages. Cell Biosci. 4, 39 10.1186/2045-3701-4-39 25228981 PMC4164960

[B109] Schön M. P. (2019). Adaptive and innate immunity in psoriasis and other inflammatory disorders. Front. Immunol. 10, 1764 10.3389/fimmu.2019.01764 31402919 PMC6676248

[B110] Schwingen J. Kaplan M. Kurschus F. C. (2020). Review-current concepts in inflammatory skin diseases evolved by transcriptome analysis: in-depth analysis of atopic dermatitis and psoriasis. Int. J. Mol. Sci. 21, 3 10.3390/ijms21030699 PMC7037913 31973112

[B111] Sertznig P. Reichrath J. (2011). Peroxisome proliferator-activated receptors (PPARs) in dermatology. Derm. Endocrinol. 3 (3), 130–135. 10.4161/derm.15025 PMC3219163 22110772

[B112] Sertznig P. Seifert M. Tilgen W. Reichrath J. (2008). Peroxisome proliferator-activated receptors (PPARs) and the human skin. Am. J. Clin. Dermatol. 9 (1), 15–31. 10.2165/00128071-200809010-00002 18092840

[B113] Silva V. Cerqueira F. Nazareth N. Medeiros R. Sarmento A. Sousa E. (2019). 1,2-Dihydroxyxanthone: effect on A375-C5 melanoma cell growth associated with interference with THP-1 human macrophage activity. Pharmaceuticals 12 (2), 85 10.3390/ph12020085 PMC6630936 31167479

[B114] Sivaranjani M. Leskinen K. Aravindraja C. Saavalainen P. Pandian S. K. Skurnik M. (2019). Deciphering the antibacterial mode of alpha-mangostin on *Staphylococcus epidermidis* RP62A through an integrated transcriptomic and proteomic approach. Front. Microbiol. 10, 150 10.3389/fmicb.2019.00150 30787919 PMC6372523

[B115] Sivaranjani M. Prakash M. Gowrishankar S. Rathna J. Pandian S. K. Ravi A. V. (2017). *In vitro* activity of alpha-mangostin in killing and eradicating *Staphylococcus epidermidis* RP62A biofilms. Appl. Microbiol. Biotechnol. 101 (8), 3349–3359. 10.1007/s00253-017-8231-7 28343241

[B116] Sivaranjani N. Rao S. V. Rajeev G. (2013). Role of reactive oxygen species and antioxidants in atopic dermatitis. J. Clin. Diagn. Res. 7 (12), 2683–2685. 10.7860/JCDR/2013/6635.3732 24551611 PMC3919411

[B117] Smith M. P. Sanchez-Laorden B. O'Brien K. Brunton H. Ferguson J. Young H. (2014). The immune microenvironment confers resistance to MAPK pathway inhibitors through macrophage-derived TNFα. Cancer Discov. 4 (10), 1214–1229. 10.1158/2159-8290.cd-13-1007 25256614 PMC4184867

[B118] Sukma M. Tohda M. Suksamran S. Tantisira B. (2011). γ-mangostin increases serotonin 2A/2C, muscarinic, histamine and bradykinin receptor mRNA expression. J. Ethnopharmacol. 135 (2), 450–454. 10.1016/j.jep.2011.03.039 21440614

[B119] Sutono T. (2013). Efficacy of *Garcinia mangostana* L. (mangosteen rind extract) to reduce acne severity. Med. J. Indonesia 22 (3), 167–172. 10.13181/mji.v22i3.586

[B120] Talevi A. (2015). Multi-target pharmacology: possibilities and limitations of the “skeleton key approach” from a medicinal chemist perspective. Front. Pharmacol. 6, 205 10.3389/fphar.2015.00205 26441661 PMC4585027

[B121] Tatiya-Aphiradee N. Chatuphonprasert W. Jarukamjorn K. (2016). *In vivo* antibacterial activity of *Garcinia mangostana* pericarp extract against methicillin-resistant *Staphylococcus aureus* in a mouse superficial skin infection model. Pharmaceut. Biol. 54 (11), 2606–2615. 10.3109/13880209.2016.1172321 27180784

[B122] Teh S. S. Ee G. C. L. Mah S. H. (2017). Evaluation of nitric oxide inhibition effect in LPS-stimulated RAW 264.7 macrophages by phytochemical constituents from *Mesua beccariana, Mesua congestiflora*, and *Mesua ferrea* . Med. Chem. Res. 26 (12), 3240–3246. 10.1007/s00044-017-2017-4

[B123] Tikhomirova L. I. Ilyicheva T. N. (2020). Preparation of biotechnological raw materials of *Iris sibirica* L. with a given content of mangiferin and antiviral activity. IOP Conf. Ser. Earth Environ. Sci. 421, 022049. 10.1088/1755-1315/421/2/022049

[B124] Tsubaki M. Takeda T. Kino T. Itoh T. Imano M. Tanabe G. (2015). Mangiferin suppresses CIA by suppressing the expression of TNF-α, IL-6, IL-1β, and RANKL through inhibiting the activation of NF-κB and ERK1/2. Am. J. Tourism Res. 7 (8), 1371–1381. PMC4568793 26396668

[B125] Vemu B. Nauman M. C. Veenstra J. P. Johnson J. J. (2019). Structure activity relationship of xanthones for inhibition of cyclin dependent kinase 4 from mangosteen (*Garcinia mangostana* L.). Int. J. Nutr. 4 (2), 38–45. 10.14302/issn.2379-7835.ijn-19-2845 31363494 PMC6667231

[B126] Wang A. Li D. Wang S. Zhou F. Li P. Wang Y. (2018a). γ-Mangostin, a xanthone from mangosteen, attenuates oxidative injury in liver via NRF2 and SIRT1 induction. J. Funct. Foods 40, 544–553. 10.1016/j.jff.2017.11.047

[B127] Wang F. Ma H. Liu Z. Huang W. Xu X. Zhang X. (2017). α-mangostin inhibits DMBA/TPA-induced skin cancer through inhibiting inflammation and promoting autophagy and apoptosis by regulating PI3K/Akt/mTOR signaling pathway in mice. Biomed. Pharmacother. 92, 672–680. 10.1016/j.biopha.2017.05.129 28582759

[B128] Wang J. J. Sanderson B. J. S. Zhang W. (2011). Cytotoxic effect of xanthones from pericarp of the tropical fruit mangosteen (*Garcinia mangostana* Linn.) on human melanoma cells. Food Chem. Toxicol. 49 (9), 2385–2391. 10.1016/j.fct.2011.06.051 21723363

[B129] Wang Q. L. Yang D. Z. Lv C. (2018b). Antiinflammatory effects of gambogic acid in murine collageninduced arthritis through PI3K/Akt signaling pathway. Mol. Med. Rep. 17 (3), 4791–4796. 10.3892/mmr.2018.8389 29328461

[B130] Wang W. M. Jin H. Z. (2020). Role of neutrophils in psoriasis. J. Immunol. Res. 2020, 3709749 10.1155/2020/3709749 32587871 PMC7293746

[B131] Watanabe K. Jose P. J. Rankin S. M. (2002). Eotaxin-2 generation is differentially regulated by lipopolysaccharide and IL-4 in monocytes and macrophages. J. Immunol. 168 (4), 1911–1918. 10.4049/jimmunol.168.4.1911 11823526

[B132] Wen J. Pei H. Wang X. Xie C. Li S. Huang L. (2014). Gambogic acid exhibits anti-psoriatic efficacy through inhibition of angiogenesis and inflammation. J. Dermatol. Sci. 74 (3), 242–250. 10.1016/j.jdermsci.2014.03.001 24685902

[B133] Wilkinson A. S. Monteith G. R. Shaw P. N. Lin C. N. Gidley M. J. Roberts-Thomson S. J. (2008). Effects of the mango components mangiferin and quercetin and the putative mangiferin metabolite norathyriol on the transactivation of peroxisome proliferator-activated receptor isoforms. J. Agric. Food Chem. 56 (9), 3037–3042. 10.1021/jf800046n 18393431

[B134] Wu S. Lu H. Bai Y. (2019). Nrf2 in cancers: a double-edged sword. Cancer Med. 8 (5), 2252–2267. 10.1002/cam4.2101 30929309 PMC6536957

[B135] Xia Y. Sun J. (2018). Synergistic inhibition of cell proliferation by combined targeting with kinase inhibitors and dietary xanthone is a promising strategy for melanoma treatment. Clin. Exp. Dermatol. 43 (2), 149–157. 10.1111/ced.13283 29168273

[B136] Xu L. Lao Y. Zhao Y. Qin J. Fu W. Zhang Y. (2015). Screening active compounds from *Garcinia* species native to China reveals novel compounds targeting the STAT/JAK signaling pathway. BioMed Res. Int. 2015, 910453 10.1155/2015/910453 26090459 PMC4450297

[B137] Xu N. Deng W. He G. Gan X. Gao S. Chen Y. (2018). Alpha- and gamma-mangostins exhibit anti-acne activities via multiple mechanisms. Immunopharmacol. Immunotoxicol. 40 (5), 415–422. 10.1080/08923973.2018.1519831 30422030

[B138] Xu X. Liu Y. Wang L. He J. Zhang H. Chen X. (2009). Gambogic acid induces apoptosis by regulating the expression of Bax and Bcl-2 and enhancing caspase-3 activity in human malignant melanoma A375 cells. Int. J. Dermatol. 48 (2), 186–192. 10.1111/j.1365-4632.2009.03946.x 19200201

[B139] Yanaka A. (2018). Role of Nrf2 in protection of gastrointestinal tract against oxidative stress. J. Clin. Biochem. Nutr. 63 (1), 18–25. 10.3164/jcbn.17-139 30087539 PMC6064821

[B140] Yun C. Chang M. Hou G. Lan T. Yuan H. Su Z. (2019). Mangiferin suppresses allergic asthma symptoms by decreased Th9 and Th17 responses and increased Treg response. Mol. Immunol. 114, 233–242. 10.1016/j.molimm.2019.07.025 31386980

[B141] Zhang B. Zhao J. Li S. Zeng L. Chen Y. Fang J. (2015). Mangiferin activates the Nrf2-ARE pathway and reduces etoposide-induced DNA damage in human umbilical cord mononuclear blood cells. Pharmaceut. Biol. 53 (4), 503–511. 10.3109/13880209.2014.927890 25380307

[B142] Zhao J. Qi Q. Yang Y. Gu H. Y. Lu N. Liu W. (2008). Inhibition of α4 integrin mediated adhesion was involved in the reduction of B16-F10 melanoma cells lung colonization in C57BL/6 mice treated with gambogic acid. Eur. J. Pharmacol. 589 (1–3), 127–131. 10.1016/j.ejphar.2008.04.063 18539272

[B143] Zhao Y. Wang W. Wu X. Ma X. Qu R. Chen X. (2017). Mangiferin antagonizes TNF-α-mediated inflammatory reaction and protects against dermatitis in a mice model. Int. Immunopharm. 45, 174–179. 10.1016/j.intimp.2017.02.014 28222357

